# Targeted Antioxidants in Exercise-Induced Mitochondrial Oxidative Stress: Emphasis on DNA Damage

**DOI:** 10.3390/antiox9111142

**Published:** 2020-11-17

**Authors:** Josh Williamson, Gareth Davison

**Affiliations:** Sport and Exercise Sciences Research Institute, Ulster University, Jordanstown Campus, Newtownabbey BT37 0QB, Northern Ireland, UK; gw.davison@ulster.ac.uk

**Keywords:** exercise, MitoQ, reactive oxygen species, mitochondrial DNA damage, cardiolipin peroxidation

## Abstract

Exercise simultaneously incites beneficial (e.g., signal) and harming (e.g., damage to macromolecules) effects, likely through the generation of reactive oxygen and nitrogen species (RONS) and downstream changes to redox homeostasis. Given the link between nuclear DNA damage and human longevity/pathology, research attempting to modulate DNA damage and restore redox homeostasis through non-selective pleiotropic antioxidants has yielded mixed results. Furthermore, until recently the role of oxidative modifications to mitochondrial DNA (mtDNA) in the context of exercising humans has largely been ignored. The development of antioxidant compounds which specifically target the mitochondria has unveiled a number of exciting avenues of exploration which allow for more precise discernment of the pathways involved with the generation of RONS and mitochondrial oxidative stress. Thus, the primary function of this review, and indeed its novel feature, is to highlight the potential roles of mitochondria-targeted antioxidants on perturbations to mitochondrial oxidative stress and the implications for exercise, with special focus on mtDNA damage. A brief synopsis of the current literature addressing the sources of mitochondrial superoxide and hydrogen peroxide, and available mitochondria-targeted antioxidants is also discussed.

## 1. Introduction

Mitochondria are recognised as multifaceted arbiters of the life and death of the cell, contributing to numerous cell networks including metabolism and biosynthetic pathways [1,2]. During oxidative phosphorylation, mitochondria generate a proton-motive force by oxidising nicotinamide adenine dinucleotide (NAD), and transporting hydrogen ions through the inner mitochondrial membrane for the purpose of adenosine 5-triphosphate (ATP) synthesis [3]. Reminiscence of its prokaryotic origin, mitochondria have retained their own genome and while the majority of mitochondrial proteins are regulated by the nuclear genome, a select number of respiratory proteins, and mitochondrial transfer and ribosomal RNAs remain encoded by mitochondrial DNA (mtDNA) [4]. These proteins are vital for the structure and function of the electron transport chain (ETC)—the series of reactions responsible for the oxidation of reduced coenzymes (NADH and FADH_2_), which are products of glucose and fatty acid catabolism.

**Figure 1:**
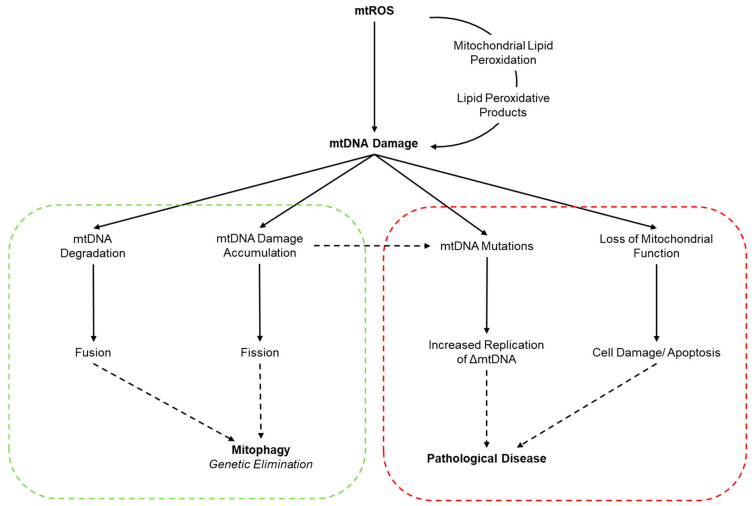
Cellular responses and downstream effects of mtDNA damage. The increased generation of ROS (and/or loss of mtDNA repair), and increase in lipid peroxidative products can potentiate mtDNA damage. Green shading represents the physiological and protective responses to mtDNA damage whereby mitochondria with excessive damage are segregated and compensated via fission and/or fusion, respectively, and are subsequently removed by mito/autophagy. Red shading indicates pathophysiological responses to mtDNA damage whereby excessive mtDNA damage can result in mtDNA mutations and eventual heteroplasmy; thus, causing a loss of mitochondrial function and potentially leading to organ pathology and disease. Adapted with permission from Van Houten et al. [30].

Regular exercise has a plethora of health benefits associated with human longevity, primarily through the prevention and management of chronic diseases [5]. These exercise-stimulated phenotypic adaptations are a consequence of acute and chronic responses, which are largely thought to be governed by redox-sensitive triggers (e.g., mitochondrial adaptation: sarcoplasmic calcium, ATP:ADP, NAD+:NADH, RONS) [3,6]. Although regular, moderately-intense exercise induces beneficial adaptations [7], sporadic and strenuous bouts of exercise incite oxidative stress due to an augmented production of reactive oxygen and nitrogen species (RONS) [8]. An accumulation of RONS impairs cell function by oxidatively modifying nucleic acids, where DNA damage and insufficient repair may potentially lead to mutagenic, clastogenic, and carcinogenic effects [9]. Currently, the majority of exercise redox research has focused on nuclear DNA damage [10,11], with a scant understanding on the relationship between exercise and mitochondrial redox dynamics in vivo. Further, evidence postulates a clear connection between mitochondrial dysfunction and disease progression [12], primarily driven by an increase in mitochondria ROS production and mtDNA damage [13,14]. This mtDNA damage can function as a redox signal thereby inducing a physiological and/or simultaneous pathological outcome as depicted in Figure 1 and briefly differentiated in Box 1.

Box 1 **The Potential Downstream Beneficial and Pathological Signals of DNA Damage.**

Under normal physiological circumstances, DNA undergoes numerous modifications from endogenous sources of RONS, compounded by exogenous physical and chemical agents (i.e., UV-irradiation, pharmacological drugs, DNA methylating agents, etc.). The current body of evidence demonstrates that high-intensity exercise is a stimulus for DNA damage. However, the potential beneficial and detrimental responses of exercise-induced DNA damage is yet to be fully elucidated. It is conceivable that an imbalance in this biological tug-of-war between DNA damage and repair can result in a pathological outcome.

**Beneficial, Health Enhancing Effects:** Regular, moderate-intensity exercise increases the production of RONS inciting damage to macromolecules, including DNA. Although complex, it has been proposed that the increase in RONS (and potentially the macromolecular damage itself) can trigger the up-regulation of transcription factors, and activate redox signals resulting in the enhanced capacity of the organism to overcome greater stress. Ultimately, over time these exercise-mediated signals trigger adaptations to RONS handling by influencing antioxidant enzyme capacity and DNA repair [15,16,17,18,19].

**Detrimental Health Effects:** Currently, the idea that exercise contributes to the development of a pathological disease is a theorem, as opposed to a hypothesis with conclusive, demonstrable evidence. Indeed, whether DNA damage is a cause or consequence of disease is an active area of research. Nevertheless, the premise involves the generation of cell DNA oxidation products such as 8-Oxo-2′-deoxyguanosine (8-Oxo-dG), which is normally removed and repaired by a DNA glycosylase (such as 8-oxoguanine DNA glycosylase—OGG1). However, unpaired 8-Oxo-dG can lead to a stalling in DNA replication forks and G > T transversion mutations [20].

To date, research examining the efficacy of systemic antioxidant treatment as a potential therapeutic to manipulate oxidative damage, and restore redox homeostasis, has produced mixed outcomes [21]. This may be due to inadequate dose and/or timing of antioxidant supplementation, poor bioavailability, heterogeneous microenvironments in which antioxidants exert their action, antioxidant status of the study population, and systemic distribution of the compound [22]. To overcome these limitations, targeted compounds have been developed for clinical applications with a specific focus on mitochondria such as MitoE [23], tiron [24,25,26], MitoQ [27,28] and MitoC [29]. In addition to the clinical perspective, the development of mitochondria-targeted compounds has allowed for a more precise exploration of in vitro/in vivo redox-sensitive processes associated with mitochondria redox dynamics.

The purpose of this review is to initially outline mitochondria superoxide and hydrogen peroxide production (O_2_·^−^/H_2_O_2_), with an emphasis on antioxidants with potential therapeutic action. Secondly, we outline the effects of exercise-mediated mitochondrial DNA damage and lipid peroxidation and the potential role of targeted antioxidant supplementation.

## 2. Mitochondrial Superoxide and Hydrogen Peroxide

Mitochondrial O_2_^−^/H_2_O_2_ are generated by electron leaks from donor redox centres of the ETC and associated metabolic enzymes causing uni- and/or bi-valent reduction of oxygen [31]. Although it is well established that mitochondrial O_2_^−^/H_2_O_2_ are generated as products of oxidative phosphorylation, the contribution of the specific site(s) responsible has always been an area of conjecture. Early calculations postulated that 2–5% of total oxygen through the electron transport chain led to O_2_·^−^ formation; later revised down to 0.15% [32,33,34]. Under contrived conditions of substrate supply and inhibition of normal electron transport, there are at least 11 mitochondrial sites that produce O_2_^−^/H_2_O_2_, which can be divided into the NADH/NAD+ and ubiquinol/ubiquinone (UQH_2_/UQ) isopotential groups: O_F_, B_F_, A_F_, P_F_, I_F_, I_Q_, II_F_, III_Qo_, G_Q_, E_F_, and D_Q_, each with different capacities [31,35].

Traditionally, the reduced flavin mononucleotide or the N-1a and N-1b iron–sulfur clusters from complex I (NADH + FMN → FMNH^−^ + NAD^+^; FMNH + O_2_ → FMN + O_2_^.−^) and the ubiquinol oxidation site of complex III (SQ^.−^ + O_2_ → Q + O_2_^.−^; Q pool- and membrane potential-dependent) are understood to be major sources of O_2_^−^/H_2_O_2_ in the respiratory chain [36,37,38]. Under appreciable substrate availability, a considerable amount of superoxide can also be generated through the tricarboxylic acid cycle dehydrogenases, when NADH/NAD+ ratios are high [39]. There is also the possibility that complex II can produce superoxide under physiological conditions (FADH^−^ + O_2_ → FAD + O_2_·^−^; [40]; however, it is likely that succinate and ubiquinol act as reducing equivalents when FADH has sufficient oxygen availability [41]. On a final point, one of the more interesting discoveries is the contribution of reverse electron transfer to mitochondrial superoxide production [42,43]. Reverse electron transfer may be stimulated by succinate, or inhibited through oxaloacetate, malonate and/or rotenone that are largely dependent on the pH gradient across the inner membrane [44,45], likely leading to modifications in ATP-sensitive potassium channels and mitochondrial O_2_^−^/H_2_O_2_ accumulation [46,47]. Although there has been significant progress in understanding superoxide production in respiratory chain complexes, conjecture still exists as to the specific mechanisms involved. For an appreciable insight to the recent discourse regarding the underlying biochemistry of superoxide production from the respiratory chain, readers are directed to the excellent reviews by Cobley [48] and Nolfi-Donegan [49].

Aside from the respiratory chain, prototypic NADPH-oxidases (NOX) are commonly categorised as a primary source of exercise-induced superoxide. More specifically, it appears NOX2 with its access to the cytosolic domain, and the localisation of NOX4 to the mitochondria are the potential suspects of exercise-mediated generation of superoxide and basal hydrogen peroxide (NOX4 only) [50,51,52]. Conjecture still remains on the role of NOX4 in mitochondria as much of the topological evidence does not differentiate between NOX4-mediated ROS release and NADH-dependent superoxide production from complex I [53]. This is further complicated by Dikalov et al. [54] and Takac et al. [55] who propose that the primary ROS released from NOX4 is hydrogen peroxide not via the classical superoxide mechanism. As a final point, while some researchers question the localisation of NOX4 in mitochondria [46], a more likely supposition is that NOX4 are exclusively located in specialised cells (kidney cortex, [56]; cardiac myocytes, [57]; vascular smooth muscle cells, [58]). Notwithstanding that the lack of antibody-specificity for direct NOX4 quantification remains problematic, it is worth considering that NOX4 may not fulfil a role in mitochondrial oxidative stress, and that other mitochondrial sources (i.e., respiratory chain complexes) can significantly exceed the production of ROS compared to NOX4 [59]. However, it is plausible to propose that extramitochondrial NOX4 acts on the redox sensitive mitochondrial protein kinase C_ε_, mitochondrial ATP-sensitive potassium channels, and/or thioredoxin 2 activity, subsequently increasing mitochondrial superoxide formation from the ETC [53,60,61]. A final contributor to mitochondrial O_2_·^−^/H_2_O_2_ accumulation are the monoamine oxidases (MAO) located in the outer mitochondrial membrane [62]. Menazza and colleagues [63] demonstrated the importance of MAO on hydrogen peroxide generation in skeletal muscle, thereby causing a loss of vitality and contractile impairments, partially related to myofibrillar protein oxidation. Contentiously, data from substrate-dependent oxidation studies suggests that MAO are potentially the most potent of all mitochondrial hydrogen peroxide producers; estimated to be 48 times more prolific than complex III (in the presence of antimycin: tyramine 45.2 µM/s versus succinate 0.95 µM/s; [64,65]). Similar to the supposition presented by Menazza et al. [63], inhibition of MAO attenuated mtDNA damage [65]. Caution is warranted regarding the contribution of MAO to hydrogen peroxide accumulation in skeletal muscle tissue due to the nanomolar concentrations of endogenous substrates [50]. Aside from the aforementioned sources, several other sites generate superoxide, including pyruvate dehydrogenase, α-ketoglutarate dehydrogenase, and transient quantal superoxide flashes [66,67,68].

To date, the major limitation with examining the topology and capacities of mitochondrial ROS is the lack of experimental and analytical approaches to empirically identify different production sites under various physiological conditions (e.g., exercise). Recently, Goncalves and colleagues [69] have pioneered the use of site-selective suppressors to inhibit O_2_^−^/H_2_O_2_ production from a single site (I_Q_ and III_Qo_) without blocking electron flow and substrate utilization, whilst not disturbing the membrane potential or energy transduction [70,71]. In a medium mimicking rest conditions, the major contributors of O_2_·^−^/H_2_O_2_ production from C2C12 myoblasts were sites II_F_, I_Q_ and I_F_ generating 24%, 23% and 20% respectively; this significantly decreased during exercise-mimicking conditions; however, site I_F_ generated 44% of total O_2_^−^/H_2_O_2_ [72]. Importantly, the concentration and type of substrate oxidised, and the concentration and redox state of the electron-donating site, are the most salient factors influencing the rate of O_2_·^−^/H_2_O_2_ release from the mitochondria [49,73]. Other contributing factors include strength of the membrane potential, local oxygen tension, electron flux, protonmotive force, ATPase activity, NADH availability, allosteric regulators, or posttranslational modifications [49,74,75].

## 3. Mitochondria-Targeted Antioxidants

Mitochondria are inherently difficult to target, as not only are there anatomical, immunological, and biochemical barriers to overcome, but the antioxidant compound must possess the physicochemical properties to cross several membranes [76,77]. Current targeting strategies to overcome these barriers (such as liposome enclosure, lipophilic cations, and targeted peptides) must exhibit several properties: (i) ability to bind to the pharmacologically active form of the compound; (ii) an efficient transport system that carries the compound to the site of its action; (iii) specific and selective targeting to the mitochondrial compartment; and (iv) release of the compound inside the mitochondrion [22,78].

Lipophilic cations are commonly utilised largely due to their ability to use the mitochondrial membrane potential (Δ*Ψ_m_*) to selectively target and accumulate in the mitochondrial matrix. In accordance with the Nernst equation ($\Delta \Psi \left(\mathrm{mV}\right)=61.5\;\times \log10\frac{c_{in}}{c_{out}}$), this allows for a 10-fold increase in diffusion and accumulation of lipophilic monovalent cations for every 61.5 mV of membrane potential [79]. Additionally, the electrochemical gradient from the plasma membrane potential (−30 to −60 mV) to the inner mitochondrial membrane potential (−150 to −180 mV) selectively targets large lipophilic cations to the mitochondria; this negative potential is specific to the mitochondria in comparison to other subcellular organelles. By using lipophilic cations, Sheu et al. [80] estimates that accumulation of specific compounds can increase 100- to 1000-fold in the mitochondria matrix.

**Figure 2:**
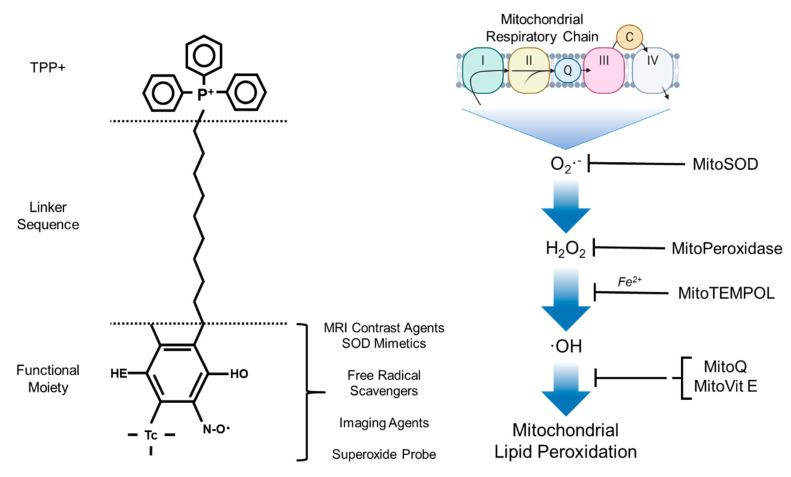
The chemical modifications and applications associated with TPP+ bioactive molecules. Adapted from Zielonka et al. [86].

Triphenylphosphonium (TTP+) is particularly effective, as it is relatively easy to introduce into a compound by displacing a leaving group by reaction with triphenylphosphine [(Ph)_3_P^+^-R]; allowing for a rapid accumulate of compound in the mitochondrial matrix [29]. The application of TPP+-conjugated bioactive molecules have been developed to deliver antioxidants, redox probes, and pharmacological agents to the mitochondria (Figure 2) [81,82,83]. These mitochondria-specific moieties are reported to react with a variety of redox modulators including hydrogen peroxide, nitric oxide, peroxynitrite, lipid peroxyl and alkoxyl radicals. With that being said, TPP+-conjugates are not without drawbacks: (i) they may be limited to low molecular weight molecules and electrically neutral chemicals; (ii) affected by mitochondrial sublocalization (i.e., the targeting of processes that occur on the outer leaflet of the inner mitochondrial membrane, the outer mitochondrial membrane, or the intermembrane space is largely limited or impossible); and (iii) high concentrations can result in depolarising of the Δ*Ψ_m_*, thus altering cell viability [22,84,85].

### 3.1. MitoQuinone, 10-(6V-Ubiquinolyl)Decyltriphenylphosphonium Bromide and 10-(6V-Ubiquinonyl)Decyltriphenylphosphonium Bromide

Coenzyme Q10 (CoQ10) is a benzoquine (2,3-dimethoxy-5 methyl-6-decaprenyl-benzoquinone) and is synthesised endogenously from the acetyl-CoA-mediated mevalonate cycle [87]. The physiological oxidoreductase properties of CoQ10 allow it to act as an electron transporter between NADH dehydrogenase and succinate dehydrogenase, and from succinate dehydrogenase to the cytochrome bc complex [87]. Selectively targeting CoQ10 to the mitochondria is limited by high lipophilicity, large molecular weight, and poor aqueous solubility; consequently, clinical trials often administer high doses (≥200 mg/day) to support prophylactic outcomes [88,89]. To overcome this, a TPP+ moiety of ubiquinol (mitoquinone [MitoQ]) comprised of a 10-carbon alkyl chain was developed by Robin Smith and Michael Murphy [90,91]. MitoQ accumulates at the matrix-facing side of the inner mitochondrial membrane where it is recycled by complex II into the active ubiquinol form (MitoQH_2_). Due to sublocalisation, MitoQ is effective against mitochondrial lipid peroxidation and peroxynitrite-induced damage by donating a hydrogen atom to oxygen-centred radicals, thereby inhibiting oxidative damage to mitochondrial lipids, proteins and DNA; this reaction generates ubisemiquinone that rapidly dismutates to ubiquinone and ubiquinol, with UQ recycled back to UQH_2_ by the mitochondrial respiratory chain (UQ + O_2_^−^ ↔ UQ^−^ + Q_2_) [92]. An additional pathway worthy of consideration is the potential of MitoQ to interact with hydroperoxyl radical species (*E°*(HOO·, H^+^/H_2_O_2_) = 1.44 V) by direct hydrogen transfer, thereby enabling superoxide to pass into biological membranes and incite oxidative damage (UQH_2_ + R·/HOO· → UQH· + RH/H_2_O_2_; 2UQH· → UQ + UQH_2_) [93]. With that being said, it is unlikely that this pathway plays a significant role in biological mediums given the slow rate reaction of UQH_2_ with R/HOO· (1−3 × 10^3^ M^−1^ s^−1^) and the greater affinity of α-tocopherol for R/HOO· (2 × 10^5^ M^−1^ s^−1^) [94]. It is perhaps more plausible that UQH_2_ exerts an antioxidant effect against R·/HOO·-mediated mitochondrial lipid peroxidation by regenerating the α-tocopheroxyl radical (αToc + R·/HOO· → αToc· + H_2_O_2_; UQH_2_ + αToc· → αToc + UQH·) [93]. In summary, it seems MitoQ has the potential to exert a number of antioxidant effects in mitochondria including: (i) oxygen-centred radical scavenging; (ii) α-tocopherol recycling; and (iii) direct reaction with superoxide; yet, under certain conditions it may exert pro-oxidant effects. On the latter, and for a comprehensive overview on the underlying biochemistry, readers are directed towards more established reviews [86,95]; however, for the purpose of brevity, in damaged membranes or close to the membrane surface, UQH_2_ can autoxidise, propagating the generation of the ubisemiquinone radical and downstream formation of superoxide and hydrogen peroxide [96,97].

### 3.2. SkQ1, Plastoquinonyl-Decyl-Triphenylphosphonium

While it is evident that MitoQ is effective against oxidative damage, there is potential for partially reduced and/or protonated intermediate forms to act as pro-oxidants through interaction with oxygen to form superoxide [92]. This presents a limitation for their therapeutic use in elevated oxidative stress scenarios [98,99]. To address this, ubiquinone was substituted for plastoquinone, thereby allowing for a 32-fold increase in the anti- versus pro-oxidant concentrations to be preferentially differentiated (MitoQ, ~300 and ~500 nM; SkQ1, ~25 and ~800 nM, respectively, [100,101]). In addition to the dampened pro-oxidant potential of SkQ1, SkQ1H_2_ (the reduced form) has a greater antioxidant capacity in comparison to MitoQ [83]. Similar to MitoQ, SkQ1 is reduced by the respiratory chain to SkQ1H_2_ and acts to prevent peroxidation of mitochondrial cardiolipin and superoxide formation [100,102]. Initially, the administration of SkQ1 was directed towards artificial lipid membranes, isolated mitochondria and animal models [101]; however, due the abundance of polyunsaturated fatty acids and exposure to atmospheric oxygen, research involving SkQ1 as a therapeutic was directed towards Phase I ophthalmic applications [103,104]. In tandem, the development of oral and injectable forms of SkQ1 and its analogue SkQR1, is a current active area of clinical research [101].

### 3.3. Szeto-Schiller Peptide-Based Strategies

In addition to the presented lipophilic-conjugated antioxidants, a series of Szeto-Schiller (SS) peptides (SS-19, SS-02, SS-31, SS-20) have been examined for their therapeutic potential as they can readily cross cell membranes and enter mitochondria due to their alternating aromatic-cationic amino acid motif [105]. Antagonistic to lipophilic-targeted molecules, SS-peptides do not rely on mitochondrial membrane potential as demonstrated on depolarised mitochondria and in the presence of an uncoupler (carbonyl cyanide-4-(trifluoromethoxy)phenylhydrazone) [106]. The mechanism of peptide mitochondrial uptake is not entirely clear, however SS-peptides are known to accumulate 1000–5000-fold and localise at the inner mitochondrial membrane via electrostatic and hydrophobic interactions [107,108].

The antioxidant properties of SS-peptides are attributed to their tyrosine or dimethyltyrosine residue allowing them to scavenge ROS effectively. It appears that the addition of dimethyltyrosine within SS-31 and SS-02 make them particularly effective at scavenging hydroxyl and peroxynitrite free radicals, with the possibility of also interacting with peroxyl radicals [109]. The protective effects of these peptides have been demonstrated in vitro in comorbidities associated with diabetes, such as retinopathy [110]. Similar in vivo observations are evident in animal models of human pathologies such as myocardial infarction [111], obesity [100] and ischemic brain injury [112]. From the literature, it appears the SS-31 moiety presents the greatest efficacy for prophylaxis due to its ability to bind to and protect against cardiolipin peroxidation by altering cytochrome c peroxidase activity [113]. To date, SS-31 has been developed as a pharmacological agent (as Bendavia/Elamipretide) and has undergone Phase I and II clinical trials (NCT02388464, NCT01572909, and NCT02367014 [114]).

### 3.4. Other Mitochondria-Targeted Compounds

The majority of research on targeted molecules has been on the aforementioned antioxidants, however, a range of mitochondria-targeted compounds have been developed using conjugated-technology with the purpose of mitigating the mitochondrial ROS cascade. The development of MitoSOD (a macrocyclic SOD mimetic) by Kelso et al. [115] allows for the selective reaction with mitochondrial superoxide above that provided by the endogenous MnSOD enzyme alone. A second SOD mimetic worthy of highlighting is MitoTEMPOL which acts to detoxify ferrous iron by oxidising ferric iron, converting the superoxide anion to water. A mitochondria targeted derivative of Ebselen (a glutathione peroxidase mimetic [MitoPeroxidase]), attenuates lipid peroxidation and prevents apoptotic cell death by clearing hydrogen peroxide [116]. Clearly, a wide variety of avenues for redox-centred research are stemming from the development of mitochondria-targeted compounds with the aim of (i) creating an array of targeted compounds for therapeutic human interventional purposes, and (ii) to further understand the significance of complex mitochondria redox signalling pathways.

## 4. Exercise and Mitochondrial Oxidative Stress

NADPH oxidase enzymes are primarily thought to drive exercise-induced O_2_^−^ production, in part, because several factors (notably ATP demand) should decrease mitochondrial O_2_^−^ production [117]. Intriguingly, while net mitochondrial O_2_^−^ production is decreased, the flavin mononucleotide of complex I continues to produce O_2_^−^ in a metabolic milieu mimicking exercise in isolated mitochondria from hind limb skeletal muscle of Wistar rats [69]. As a result, the persistent nature of mitochondrial O_2_·^−^ production means a low rate of net production can cause damage over time. Furthermore, it could be hypothesised that as exercise intensity increases, H_2_O_2_ production from the electron transport chain decreases while simultaneously increasing the ability of the mitochondria to sequester extramitochondrial H_2_O_2_; thereby increasing the likelihood of Fenton-mediated reactions, and subsequent mtDNA damage. In addition to our limited understanding of the effects of exercise on mitochondrial oxidative stress, the use of non-specific, pleiotropic antioxidants makes it difficult to draw any precise mechanistic or practical conclusions. To that end, the following sections (i) detail our current knowledge and understanding of exercise-induced perturbations to mitochondrial oxidative stress, and (ii) offers potential implications for the use of targeted antioxidant supplementation.

Fogarty and colleagues [118] were the first to demonstrate the impact of exercise on mtDNA by showing that isolated and maximal contractions increased mitochondrial 8-hydroxy-2-deoxyguanosine concentration in vastus lateralis muscle tissue. More recently, we have demonstrated an increase in global mtDNA damage in peripheral blood mononuclear cells and skeletal muscle (as quantified using LA-qPCR) following high-intensity intermittent exercise [119]. It is highly likely the observed mtDNA damage originated from O_2_^−^/H_2_O_2_-derived mechanisms, stemming from their ability to react with accessible transition metals to produce damaging hydroxyl free radical species via Fenton chemistry (H_2_O_2_ + Fe^2+^ → Fe^3+^ + ^−^OH + ·OH [*k* ~ 76 M^−1^ s^−1^]; H_2_O_2_ + Cu^+^ → Cu^2+^ + ^−^OH + ·OH [*k* ~ 4.7 × 10^3^ M^−1^ s^−1^]) [120]. As a result, the hydroxyl free radical appreciably reacts with DNA bases at diffusion-controlled rates (*k* ~ 5–8 × 10^9^ M^−1^ s^−1^ for guanine [121]); potentially generating other end products, which can further propagate oxidative damage [122]. Interestingly, a recent supposition proposes that the carbonate radical anion as opposed to the hydroxyl free radical is the predominate radical formed from the Fenton reaction (Fe^II^(CO_3_)(OOH)(H_2_O)_2_ → Fe^III^(OH)_3_(H_2_O) + CO_3_·^−^) [123,124]. Given the reduction potentials of both the hydroxyl and carbonate radicals (2.4 and 1.6 V, respectively), it is not surprising that 2′-deoxyguanosine is a common outcome for these potent one-electron oxidants. With that being said, the biological relevance and implications of the carbonate radical in Fenton-mediated chemistry and subsequent oxidative damage is yet to be ascertained.

Historically, it was understood that exercise-induced superoxide predominately originated from the respiratory chain [32]; however, recent estimations suggest greater concentrations of mitochondrial superoxide are produced during State 4 respiration (i.e., basal) in comparison to State 3 (i.e., exercise). Additionally, although the intricacies of NADPH oxidases are complicated, the mitochondrial NOX4 is constitutively active and likely contributes to basal O_2_·^−^/H_2_O_2_ production in skeletal muscle tissue. Furthermore, it appears evident that contraction-induced superoxide production is largely derived from non-mitochondrial sources; specifically NOX2 due to agonist activation (e.g., angiotensin II, cytokines, and mechanical stress) [125,126,127]. Notwithstanding the potential for delayed mitochondrial redox dynamics, collectively, the apparent decrease in exercise-mediated mitochondrial O_2_^−^/H_2_O_2_ production as suggested by Goncalves et al. [69], in tandem with the observed increase in mtDNA damage [118,119], presents a major paradoxical juxtaposition of our understanding of mitochondrial redox dynamics in the context of exercise. While much of the topological research of mitochondrial O_2_·^−^/H_2_O_2_ generation has focused on the specific complexes of the respiratory chain [69,72], others have shown correlations between mitochondrial H_2_O_2_ generation and P^66Shc^-FOXO3a expression in an intensity-dependent manner in the skeletal muscle of mice [128,129]. With that being said, the narrative that P^66Shc^ is responsible for the accumulation of mitochondrial H_2_O_2_ and consequential mitochondrial oxidative damage as a function of exercise, albeit biologically plausible, is unfounded; especially in the in/ex vivo human model.

An additional proposition worth discussing is for the exercise-induced H_2_O_2_ to be a nexus point of nuclear and mitochondrial redox signalling [130]. The mitochondria play a dichotomous role in the production and degradation of H_2_O_2_ [131,132]; however, they also have the potential to quench extramitochondrial or cytosolic H_2_O_2_ when the rate of O_2_·^−^ release from the respiratory chain is low (i.e., during exercise [69,72]) and via the glutathione and thioredoxin systems [133,134,135]. Murphy [136] outlines that mitochondrial H_2_O_2_ has a large capacity for diffusion, and is hypothesised to play distinct roles in downstream physiological signalling responses, including post-translational modifications [131,137]. It is clear that low-to-moderate intensity/duration exercise exerts ROS-mediated triggers for adaptation signalling with minimal DNA damage [138]; however, as exercise intensity increases, so too does damage to DNA [139]. It could be hypothesised that as exercise intensity increases, mitochondria act as an ‘antioxidant’ per se by sequestering cytosolic H_2_O_2_ and dissipating it through the catalase, glutathione, and thioredoxin antioxidant systems [140]. This ability of the mitochondria to quench cytosolic H_2_O_2_ has been demonstrated by others [133,141]; however, it appears to be largely dependent on (i) the rate of mitochondrial hydrogen peroxide production, and (ii) the rate of mitochondrial H_2_O_2_ degradation [135].

## 5. Exercise-Induced Mitochondrial Oxidative Stress and the Role of Targeted Compounds

Research examining exercise-induced oxidative stress and antioxidants largely focuses on two primary outcomes: (i) induction/attenuation of macromolecular damage, and (ii) the redox signalling of exercise adaptation [10,142]. Over the last two decades there has been a paradigm shift in our understanding of exercise-induced oxidative stress and antioxidants; firstly, the ability of reactive species and antioxidants to improve exercise performance, and secondly, the implications of antioxidant supplementation on exercise adaptation [6,143]. Given the role of oxidative stress and antioxidant supplementation on exercise redox biology, and the emerging importance of redox-mediated retro/antero-grade signalling (i.e., mitochondrial-nuclear communication), our understanding of the role of exercise-induced perturbations to mitohormesis and mitochondrial-targeted compounds is limited. In this section, we attempt to propose the role of mitochondrial-targeted compounds on the potential implications for exercise and oxidative damage to mitochondrial macromolecules.

### 5.1. Mitochondrial DNA Damage

Mitochondria contain a polyploid, 16,569 base pair circular genome which can be categorised as homo or heteroplasmy depending on the sequencing of multiple copies within the cell [144]. Maintaining the integrity of the mitochondrial genome is important to prevent irreversible loss or modification of its coded information, which is particularly detrimental in postmitotic tissue [145]. Free radical oxidants are known to produce an array of DNA oxidative modification, which may be potentiated by the mitochondrial matrix microenvironment, and until very recently [146,147], methodological and analytical techniques have lacked the specificity and sensitivity to detect the board spectrum of mtDNA lesions [148]. Mitochondrial DNA is particularly susceptible to oxidative attack, resulting from the close proximity of the genome (and abundance of cardiolipin and secondary peroxidative products) to multiple sources of mitochondrial superoxide, lack of protective histone proteins, and limited capacity for repair [39,149,150,151]. Consequently, genetic information is more tightly packed, resulting in higher relative levels of oxidative damage in comparison to the nuclear genome; thus amplifying the potential downstream detrimental and mutational effects [145]. For one, correlations between mitochondrial ROS and mtDNA strand breaks suggest that greater DNA damage has the potential to incite a greater formation of mtROS as impaired mtDNA translation leads to mitochondrial uncoupling with secondary increases in mtROS formation [152]. To elaborate, one of the most common oxidative modifications, 8-oxo-deoxyguanosine, is a potential mutagenic lesion, and its accumulation is directly correlated with the development of pathological processes [153,154]. Finally, selective mtDNA variants are an underlying factor in mitochondrial diseases and other pathologies; thus clarity on the downstream beneficial/detrimental consequences of oxidative mtDNA damage is warranted [155].

Beyond the observable increase in mtDNA damage following exercise, our current understanding of the effects of this damage, on mitochondrial redox dynamics (and indeed signalling) is unknown. The observations by Fogarty and colleagues [118] provide tentative evidence for a Fenton-mediated mechanism of hydroxyl attack on DNA; however, it is worth noting that these analyses were performed on isolated mitochondria from muscle tissue which have the potential to incite artefactual oxidation. To help address this, we recently used long amplicon-qPCR to ascertain the effect of high-intensity intermittent exercise on mitochondrial DNA. Following 4 × 4-minute bouts of exercise, we observed an increase in mtDNA damage (lymphocytes and muscle tissue), and detectable changes in lipid peroxidation and the ascorbyl free radical; some of which was mitigated by chronic MitoQ supplementation (20 mg/day for 3 weeks [119]). In both instances, the exercise-induced mtDNA damage is likely initiated by: (i) inhibiting repair; (ii) increasing H_2_O_2_ production; and/or (iii) increasing the labile redox-active transition metal pool via hydroxyl (and potentially peroxynitrite-derived) radicals [120,122].

The role of exercise-induced mtDNA damage is enigmatic, and although generally considered harmful, the current lack of understanding associated with mtDNA allows scope for oxidative damage adducts to initiate or act as beneficial signals. One proposition is for oxidative modifications to DNA (e.g., 8-oxo-deoxyguanosine) to trigger the upregulation of repair genes and other genes that need to respond to the perturbations in cellular and/or mitochondrial redox state [124]. Additionally, DNA repair pathways help prevent heteroplasmy and maintain genome integrity; however, conjecture still surrounds whether these repair enzymes pre-exist in mitochondria at the time of damage or translocate into the mitochondria in response to damage signals [156]. These antero/retrograde signalling processes between the mitochondria and nucleus respond to a loss of mtDNA, damage accumulation, and oxidative stress trigger signals, ultimately dictating downstream organelle and/or cellular adaptations [157,158,159]. Firstly, mtDNA damage causes a rise in calcium concentration, leading to an activation of protein kinase C, c-Jun N-terminal kinase/p38 and Ca2+/calmodulin-dependent protein kinases that are not only linked to post-translational modifications to DNA repair proteins, but also are contributing factors in the signal transduction pathway of mitochondrial biogenesis [160]. Secondly, NAD^+^ is a critical substrate for the PARP and SIRT families which deacetylate and activate a complex network of proteins associated with the DNA damage-repair response and cellular adaptation (i.e., PGC1α, Ku70, NF-κβ, and NIF1α [161]). Finally, p53 and ataxia telangiectasia mutated are redox-sensitive and can function to phosphorylate and activate AMP-activated protein kinase, subsequently regulating the activity of the previously mentioned anterograde signalling triggers (SIRT1, PGC-1α, and HIF-1α). To conclude, while exercise increases the accumulation of RONS which trigger the activation of downstream transcription factors it also appears that oxidative- and mitochondrial-stress (including mtDNA damage) modulate redox-sensitive triggers (Ca^2+^, ATP:AMP, NAD^+^:NADH) which signal for adaptations associated with mitochondrial biogenesis, metabolism, and maintenance of genome integrity [156,160,161]. Interestingly, it has been hypothesised that oxidative stress activates mitochondrial DNA methyltransferases, which promote epigenetic modifications responsible for mitochondrial transcription and promote the accumulation of mitochondrial and nuclear 8-hydroxy-2′-deoxyguanosine [162,163].

### 5.2. Mitochondrial Lipid Peroxidation

The mitochondria contain a high prevalence of lipids such as anionic cardiolipin, phosphatidylethanolamine, and phosphatidylcholine, which are vulnerable to oxidative attack at varied rates initiated by RONS (e.g., hydroxyl radicals, superoxide, peroxyl radical, nitric oxide, peroxynitrite or nitrogen dioxide) [164]. These accumulated phospholipids, in combination with the high rate of oxygen utilisation and peroxidative catalysts (i.e., heme and non-heme iron), potentiate oxidative damage to mitochondrial lipids which can contribute to mitochondrial dysfunction [165], the pathogenesis of inflammation [166], and numerous age-related diseases [167,168]. In addition to this direct oxidative insult, lipid peroxidation products may contribute to DNA damage, thus propagating the oxidative damage cascade; this may partially explain the elevated mitochondrial mutations observed in diseases and inactive respiratory complexes [169,170]. Although there are a variety of peroxidative targets, it appears that cardiolipin is a central component of mitochondrial oxidative stress. For one, it is the most sensitive constituent of the inner mitochondrial membrane [100]. Secondly, cardiolipin acts as an anchor for cytochrome c and mtDNA (specifically during replication), of which peroxidative attack results in cytochrome c release, accumulation of apoptosis factors, and propagation of the peroxidation cascade [171,172,173]. Finally, loss of cardiolipin results in inhibition of respiratory chain complexes, H^+^-ATP-synthase, ATP/ATP-antiporter subsequently leading to an increase in permeability of the inner mitochondrial membrane and, as a consequence, resulting in the collapse of Δψ, swelling of matrix, disruption of the outer mitochondrial membrane and release of cytochrome c into the extramitochondrial space [174].

It is worth highlighting the significance of exercise-mediated lipid peroxidation in relation to DNA damage, as these biological scenarios are intertwined [139]. Briefly, the formation of a lipid radical can give rise to various lipid peroxidation products including lipid hydroperoxides, alkoxyl radicals, and aldehydes [175]. Aldehydes in particular can react with DNA, and it is known that malondialdehyde can lead to mutagenic insertions, deletions, and base pair substitutions, and can react with nitrogen to form the malondialdehyde-2′-deoxyguanosine adduct. The compound 4-hydroxy-2-nonenal interacts with DNA including Michael addition of the N2-amino group of deoxyguanosine resulting in the formation of the γ-hydroxy-1,N2-propano-2′-deoxyguanosine adduct [175,176,177]. We have previously shown that exercise-induced DNA damage is associated with lipid-derived alkoxyl free radical production, and this occurred in the presence of raised lipid hydroperoxides [139]. Thus, given that mitochondrially-targeted antioxidants are effective at scavenging the by-products of lipid peroxidation [80,170,178], attenuating exercise-induced lipid peroxidation may decrease the harmful effects of downstream DNA damage.

Initially, much of the research focused on MitoQ due to its ability to (i) accumulate in mitochondria in a Δψ-dependent manner; (ii) act as an effective antioxidant against hydroxyl-mediated mitochondrial lipid peroxidation; (iii) facilitate cell survival at much lower concentrations than CoQ or α-tocopherol; and (iv) prolong longevity at high O_2_ tension [82,100,179]. However, the ability of MitoQ to act as an antioxidant changes to a prooxidant when MitoQ concentration is increased (e.g., 0.5–1 µM versus 2 µM on hydrogen peroxide production) [100,102,104]. As a result, derivatives of targeted quinone compounds were prioritised due to the larger antioxidant versus prooxidant window. In a series of seminal experiments, Skulachev and colleagues [83] characterised cationic antioxidants within the propagation phase of lipid peroxidation (LO_2_• + QH_2_ → LOOH + QH• vs. LO_2_• + LH → LOOH + L•). They suggested the antioxidant effects of plastoquinone-derived compounds against mitochondrial lipid peroxidation are two-fold: (i) direct quenching of radical intermediates of cardiolipin peroxidation ([1] LO2· + SkQH2 → LOOH + SkQ−·, [2] O2-z· + SkQ → O2 + SkQ−·) and (ii) potentiating mild uncoupling by mediating the transport of fatty acid anions ([3] (RCOO-)_in_SkQ(RCCO-)_out_, [4] (LOOH)_in_SkQ(LOOH)_out_); however, this latter mechanism may be contentious in MitoQ or SkQ1 in comparison to dodecyltriphenylphosphonium (C_12_TPP).

### 5.3. Implications for Exercise

The initial proposition that ROS are inherently detrimental to health suggests that synthetic antioxidants may be used to reduce oxidative damage, thus offering a potential therapy to pathological outcomes. Later, the shift in paradigm uncovered the paradoxical beneficial roles of ROS in pathophysiology. This curvilinear relationship between ROS and oxidative stress also appears to be true for the use of antioxidant supplementation. Currently, it is apparent that antioxidant supplementation seems to play dichotomous roles regarding exercise: (i) the improvement of cellular redox state and decreased modifications to DNA, lipids, and proteins via the neutralisation of exercise-induced ROS generation leading to possible impairments in the adaptive changes associated with chronic exercise [7,180,181,182], and (ii) based on the original hypothesis by Reid [183], exogenous antioxidant supplementation can sustain muscle contraction and exercise performance [138,184,185]. Nevertheless, this juxtaposition of systemic antioxidants (whether sole or in combination) and the implications for exercise is beyond the scope of this review, and readers are directed towards the more nuanced works of Mason et al. [186], Merry & Ristow [187], and Reid [188].

The role of mitochondria-targeted antioxidants on both the acute and chronic responses of exercise redox physiology is an active area of research. Although N-acetylcysteine has been shown to be particularly effective in improving exercise fatigue tolerance [189,190]—likely through the regulation of ROS—mitochondrial-targeted antioxidants don’t appear to have the same efficacy. Interestingly, Cheng and colleagues [81] demonstrated that although effective at sequestering mitochondrial superoxide, SS-31 had no impact on fatigue in mouse muscle, suggesting that ROS-induced fatigue is mediated by the cytosolic ROS challenge, possibly resulting from modifications to cellular Ca^2+^ handling. Additionally, Siegel and colleagues [191] who reported a restoration in age-related decline in mitochondrial coupling efficiency, phosphorylation capacity, and PCr/ATP ratio, translating to an enhanced exercise fatigue resistance and exercise capacity. Although this was demonstrated in an animal model, it supports the notion that targeted antioxidants may have therapeutic benefits in mitochondrial dysfunction scenarios. Indeed, our recent work [119] failed to detect any changes in basal mtDNA damage following chronic MitoQ supplementation; however, it did reduce exercise-induced mtDNA damage. We propose that the use of MitoQ may be limited in healthy individuals with low basal oxidative damage [11,192]; however, targeted supplementation may show efficacy in conditions characterised by chronic oxidative stress and/or mitochondrial dysfunction [193,194,195].

Although the human evidence on MitoQ is limited, the clinical evidence appears to confirm this hypothesis with promising results of MitoQ therapy in Parkinson’s disease [196], Hepatitis C-induced liver damage [197], and vascular dysfunction [28]. More recently, other researchers have demonstrated a dose-dependent increase in exercise performance in patients with mitochondrial myopathy following SS-31 administration, likely through modulating ATP synthesis [198]. Although there is a putative mechanism and safety with targeted antioxidants (specifically MitoQ and SS-31), and the emerging evidence suggests a therapeutic potential for clinical applications, it doesn’t appear to translate to any measure of exercise performance in healthy individuals [199]. It seems the premise for antioxidant supplementation as an ergogenic aid for improving exercise performance and fatigue resistance is largely driven through the redox state of the cytosolic environment and systemic antioxidant status as opposed to the mitochondrial compartment. With that being said, it is evident that mitochondria-targeted compounds act on various parameters of muscle, such as mitochondrial function and capacity, contractile function, and insulin sensitivity [200]. A call for future research is warranted to elucidate whether the: (i) promising results achieved in the animal model are aligned to humans, (ii) acute reduction in mitochondrial ROS translates to an ergogenic effect through the consumption of single or combinations of mitochondria-targeted antioxidants, and (iii) beneficial effects associated with targeted antioxidants are exclusively applicable to clinical populations, or in individuals with elevated basal mitochondrial oxidative stress.

The acute exercise responses to antioxidants are only one face of the coin: Merry & Ristow [187] and Reid [188] have published comprehensive reviews discussing whether synthetic antioxidant supplementation hampers the chronic responses of exercise in skeletal muscle through the blunting of redox-signalling pathways. As a general consensus, it seems there is a lack of conclusive evidence for most antioxidant compounds regarding exercise-related redox signalling and the subsequent adaptations in skeletal muscle and vascular function [186]; indeed, this is of course more applicable for targeted-antioxidant applications. Shill and colleagues [199] are the only group to investigate the effects of chronic mitochondria-targeted antioxidant supplementation (MitoQ; 10 mg/day for 3 weeks) on exercise training-induced adaptations and performance outcomes in healthy young men, with no observable effects on V^.^O_2max_, oxidative capacity, or circulating angiogenic cells reported. This supports the hypothesis that antioxidant supplementation may only be beneficial in populations where chronic oxidative stress and perturbed redox homeostasis is likely to be present. Additionally, Min et al. [201], (albeit within the animal model) demonstrated that treatment with SS-31 protected skeletal muscle against the production of mitochondrial ROS, mitochondrial uncoupling, oxidative damage, and inactivity-induced atrophy. It was concluded that mitochondrial ROS and subsequent oxidative damage triggered upstream alterations in cytosolic free Ca^2+^, activating calpain and capase-3, in turn causing skeletal muscle atrophy. This provides a tentative hypothesis for the beneficial blunting of adaptation signalling through mitochondrial ROS.

**Figure 3:**
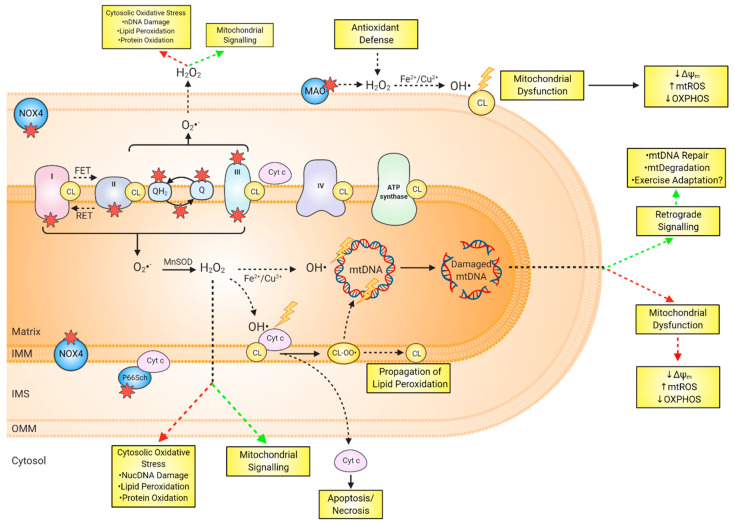
Overview of mitochondrial redox dynamics and the complexities associated with examining mitochondrial O_2_^−^/H_2_O_2_ generation and downstream consequences. Potential sources of mitochondrial O_2_^−^/H_2_O_2_ (as indicated by the red star) include complex I, complex II, complex III, mGPDH, ETF/ETFQO, DHODH, MAO, NOX4 and P66Sch allow for the release of O_2_·^−^/H_2_O_2_ into the inner mitochondrial space, or the mitochondrial matrix. Mitochondrial superoxide is dismutated to hydrogen peroxide by Mn-SOD. Hydrogen peroxide can act as an extramitochondrial signal transducer, or in the presence of liable transition metals can undergo Fenton (Fe^II^_aq_+H_2_O_2_) chemistry causing oxidative damage to mtDNA and cardiolipin. Damage to mtDNA can (i) activate signal transduction associated with mtDNA repair, and/or (ii) induce mitochondrial dysfunction, subsequently leading to the propagation of mitochondrial O_2_·^−^/H_2_O_2_ and further oxidative insult. Depending on the sublocalisation, cardiolipin oxidation can result in, (i) the release of cytochrome c to the cytosol, signalling for apoptosis/necrosis, (ii) the generation of lipid peroxidation by-products capable of attacking adjacent cardiolipin molecules and damaging mtDNA, and (iii) respiratory chain complex and mitochondrial dysfunction, thus further propagating the mitochondrial oxidative cascade. It is difficult to ascertain the role exercise plays in the schematic, not to mention the addition of mitochondrial-targeted antioxidants. Targeted supplementation may attenuate the detrimental effects associated with mitochondrial O_2_·^−^/H_2_O_2_ generation (as outlined by the red arrows) while acting independent to the beneficial effects of exercise adaptation and genome maintenance (as outlined by the green arrows). Abbreviations: OH**·**, hydroxyl free radical; CL, cardiolipin; CL-OO**·**, cardiolipin-peroxyl radical; Cyt C, Cytochrome C; DHODH, dihydroorotate dehydrogenase; ETFQOR, electron transferring flavoprotein:ubiquinone oxidoreductase; Fe^2+^/Cu^2+^, iron/copper; FET, forward electron transfer; H_2_O_2_, hydrogen peroxide; IMM, inner mitochondrial membrane; IMS, intermembrane space; OMM, outer mitochondrial membrane; MAO; monoamine oxidase; mGPDH, mitochondrial glycerol-3-phosphate dehydrogenase; MnSOD, manganese superoxide dismutase; NOX4, NADPH oxidase isoform 4; O_2_·^−^, superoxide; OMM, outer mitochondrial membrane; Q, Ubiquinone; QH2, Ubiquinol; RET, reverse electron transfer; nDNA, nuclear DNA.

Several lines of research suggest that mitochondrial ROS are implicated in retrograde signalling to the nuclear genome to stimulate a biogenic response, possibly to replace oxidatively damaged mitochondrial proteins or DNA [202,203]. Indeed, mitochondrially-produced ROS contribute to phosphatase inactivation and insulin receptor autophosphorylation [204,205], in addition to the phosphorylation of phosphatidylinositol 3 kinase and downstream activation of AKT signalling; a process core to cell survival and mitochondrial biogenesis [206,207]. It is evident that acute stimulation with exogenous oxidants activates mitochondrial biogenesis [208,209], either through PGC-1α- or PI3K/AKT-mediated pathways [210]. The redox-activated PGC-1α is also associated with the expression of antioxidant genes such as Prx3, Prx5, Trx2, TrxR2, catalase, SOD2, and GPx-1 [211,212]. Additionally, mtDNA damage signals for mtDNA degradation as an important damage response mechanism to maintain genomic integrity [213,214,215]. Supporting evidence suggests that the mtDNA degradation pathway is more important in reducing the abundance of oxidative DNA lesions and mutagenesis than the inferred lack of mtDNA repair pathways reported in earlier studies [216,217]. Collectively, there is ample evidence that mitochondrial ROS are implicated in numerous signalling pathways such as apoptosis, autophagy/mitophagy, necrosis, and pyroptosis through activation of multiple core signalling factors including AMP-activated protein kinase, mitogen-activated protein kinase kinase kinase/mitogen-activated protein kinase 8, Ca2+ kinase II, cyclic adenosine monophosphate response element binding protein, and nuclear factor-κβ pathways [218,219,220]. As recently highlighted by Burtscher and colleagues [221], there is an urgency to examine whether a reduction in mtDNA damage following targeted supplementation is independent of beneficial molecular and physiological exercise adaptations under conditions of reduced redox signalling, and if there are consequences, for example, on muscle fatigue and muscle damage. Examining modifications to mitochondrial redox dynamics are complex (as outlined in Figure 3) due to the interplay between mitochondrial ROS generation, oxidised mtDNA and lipids, and downstream signaling; however, the proposed use of mitochondria-targeted compounds to alter this network presents a poignant call for carefully planned research.

## 6. Conclusions and Future Perspectives

Mitochondrial DNA damage in the context of exercise is an exciting area of emerging research with many avenues to be explored. To the authors knowledge, only two studies have examined the effects of exercise on mtDNA damage, highlighting that research examining the role of mitochondria-targeted antioxidants on exercise-mediated changes to mitochondrial oxidative dis/eustress is very much in its infancy. As such, there is a need for future research to ascertain the effects of exercise on mtDNA modification and redox dynamics. The authors propose that further work should consider (i) whether mitochondrial oxidative damage may be a molecular trigger for exercise-related cell adaptations, (ii) the role of targeted antioxidants in the regulation of exercise performance, (iii) whether the regulation of mitochondrial redox status through targeted antioxidants is exclusively beneficial to clinical populations with elevated basal mitochondrial oxidative stress. To this end, we hope that this narrative stimulates further research aligned to exercise-induced mtDNA damage and targeted antioxidants across healthy and disease models.

## References

[1] Chan D.C. (2012). Fusion and fission: Interlinked processes critical for mitochondrial health. Annu. Rev. Genet..

[2] Hoitzing H., Johnston I.G., Jones N.S. (2015). What is the function of mitochondrial networks? A theoretical assessment of hypotheses and proposal for future research. BioEssays.

[3] Huertas J.R., Casuso R.A., Agustín P.H., Cogliati S. (2019). Stay fit, stay young: Mitochondria in movement: The role of exercise in the new mitochondrial paradigm. Oxid. Med. Cell. Longev..

[4] Taanman J.W. (1999). The mitochondrial genome: Structure, transcription, translation and replication. Biochim. Biophys. Acta-Bioenerg..

[5] Booth F.W., Roberts C.K., Thyfault J.P., Ruegsegger G.N., Toedebusch R.G. (2017). Role of inactivity in chronic diseases: Evolutionary insight and pathophysiological mechanisms. Physiol. Rev..

[6] Margaritelis N.V., Paschalis V., Theodorou A.A., Kyparos A., Nikolaidis M.G. (2020). Redox basis of exercise physiology. Redox Biol..

[7] Ristow M., Zarse K., Oberbach A., Klöting N., Birringer M., Kiehntopf M., Stumvoll M., Kahn C.R., Blüher M. (2009). Antioxidants prevent health-promoting effects of physical exercise in humans. Proc. Natl. Acad. Sci. USA.

[8] Packer L., Cadenas E., Davies K.J.A. (2008). Free radicals and exercise: An introduction. Free Radic. Biol. Med..

[9] Sharma A., Singh K., Almasan A. (2012). DNA Repair Protocols. Methods Mol. Biol..

[10] Tryfidou D.V., McClean C., Nikolaidis M.G., Davison G.W. (2020). DNA Damage Following Acute Aerobic Exercise: A Systematic Review and Meta-analysis. Sport. Med..

[11] Fisher-Wellman K., Bloomer R.J. (2009). Acute exercise and oxidative stress: A 30 year history. Dyn. Med..

[12] Chakrabarty S., Kabekkodu S.P., Singh R.P., Thangaraj K., Singh K.K., Satyamoorthy K. (2018). Mitochondria in health and disease. Mitochondrion.

[13] Druzhyna N.M., Wilson G.L., LeDoux S.P. (2008). Mitochondrial DNA repair in aging and disease. Mech. Ageing Dev..

[14] Richter C. (1995). Oxidative damage to mitochondrial DNA and its relationship to ageing. Int. J. Biochem. Cell Biol..

[15] Collins A.R., Gaivão I. (2007). DNA base excision repair as a biomarker in molecular epidemiology studies. Mol. Aspects Med..

[16] Mota M.P., Peixoto F.M., Soares J.F., Figueiredo P.A., Leitão J.C., Gaivão I., Duarte J.A. (2010). Influence of aerobic fitness on age-related lymphocyte DNA damage in humans: Relationship with mitochondria respiratory chain and hydrogen peroxide production. Age (Omaha).

[17] Cash S.W., Beresford S.A.A., Vaughan T.L., Heagerty P.J., Bernstein L., White E., Neuhouser M.L. (2014). Recent physical activity in relation to DNA damage and repair using the comet assay. J. Phys. Act. Heal..

[18] Soares J.P., Silva A.M., Oliveira M.M., Peixoto F., Gaivão I., Mota M.P. (2015). Effects of combined physical exercise training on DNA damage and repair capacity: Role of oxidative stress changes. Age (Omaha).

[19] Moreno-Villanueva M., Kramer A., Hammes T., Venegas-Carro M., Thumm P., Bürkle A., Gruber M. (2019). Influence of acute exercise on dna repair and parp activity before and after irradiation in lymphocytes from trained and untrained individuals. Int. J. Mol. Sci..

[20] Wu J., Sturla S.J., Burrows C.J., Fleming A.M. (2019). Impact of DNA Oxidation on Toxicology: From Quantification to Genomics. Chem. Res. Toxicol..

[21] Hajhashemi V., Vaseghi G., Pourfarzam M., Abdollahi A. (2010). Are antioxidants helpful for disease prevention?. Res. Pharm. Sci..

[22] Apostolova N., Victor V.M. (2015). Molecular strategies for targeting antioxidants to mitochondria: Therapeutic implications. Antioxidants Redox Signal..

[23] Mao G., Kraus G.A., Kim I., Spurlock M.E., Bailey T.B., Zhang Q., Beitz D.C. (2010). A mitochondria-targeted vitamin E derivative decreases hepatic oxidative stress and inhibits fat deposition in mice. J. Nutr..

[24] Krishna C.M., Liebmann J.E., Kaufman D., DeGraff W., Hahn S.M., McMurry T., Mitchell J.B., Russo A. (1992). The catecholic metal sequestering agent 1,2-dihydroxybenzene-3, 5-disulfonate confers protection against oxidative cell damage. Arch. Biochem. Biophys..

[25] Silveira L.R., Pereira-Da-Silva L., Juel C., Hellsten Y. (2003). Formation of hydrogen peroxide and nitric oxide in rat skeletal muscle cells during contractions. Free Radic. Biol. Med..

[26] Fang Y., Hu X.H., Jia Z.G., Xu M.H., Guo Z.Y., Gao F.H. (2012). Tiron protects against UVB-induced senescence-like characteristics in human dermal fibroblasts by the inhibition of superoxide anion production and glutathione depletion. Australas. J. Dermatol..

[27] Lowes D.A., Thottakam B.M.V., Webster N.R., Murphy M.P., Galley H.F. (2008). The mitochondria-targeted antioxidant MitoQ protects against organ damage in a lipopolysaccharide-peptidoglycan model of sepsis. Free Radic. Biol. Med..

[28] Rossman M.J., Santos-Parker J.R., Steward C.A.C., Bispham N.Z., Cuevas L.M., Rosenberg H.L., Woodward K.A., Chonchol M., Gioscia-Ryan R.A., Murphy M.P. (2018). Chronic supplementation with a mitochondrial antioxidant (MitoQ) improves vascular function in healthy older adults. Hypertension.

[29] Finichiu P.G., Larsen D.S., Evans C., Larsen L., Bright T.P., Robb E.L., Trnka J., Prime T.A., James A.M., Smith R.A.J. (2015). A mitochondria-targeted derivative of ascorbate: MitoC. Free Radic. Biol. Med..

[30] Van Houten B., Hunter S.E., Meyer J.N. (2016). Mitochondrial DNA damage induced autophagy, cell death, and disease. Front. Biosci.-Landmark.

[31] Wong H.S., Dighe P.A., Mezera V., Monternier P.A., Brand M.D. (2017). Production of superoxide and hydrogen peroxide from specific mitochondrial sites under different bioenergetic conditions. J. Biol. Chem..

[32] Boveris A., Chance B. (1973). The mitochondrial generation of hydrogen peroxide. General properties and effect of hyperbaric oxygen. Biochem. J..

[33] Loschen G., Azzi A., Richter C., Flohe L. (1974). Superoxide Radicals as Precursors of Mitochondrial Hydrogen Peroxide. FEBS Lett..

[34] St-Pierre J., Buckingham J.A., Roebuck S.J., Brand M.D. (2002). Topology of superoxide production from different sites in the mitochondrial electron transport chain. J. Biol. Chem..

[35] Muller F.L., Liu Y., Van Remmen H. (2004). Complex III releases superoxide to both sides of the inner mitochondrial membrane. J. Biol. Chem..

[36] Pryde K.R., Hirst J. (2011). Superoxide is produced by the reduced flavin in mitochondrial complex I: A single, unified mechanism that applies during both forward and reverse electron transfer. J. Biol. Chem..

[37] Kushnareva Y., Murphy A.N., Andreyev A. (2002). Complex I-mediated reactive oxygen species generation: Modulation by cytochrome c and NAD(P)+ oxidation-reduction state. Biochem. J..

[38] Bleier L., Dröse S. (2013). Superoxide generation by complex III: From mechanistic rationales to functional consequences. Biochim. Biophys. Acta-Bioenerg..

[39] Brand M.D. (2016). Mitochondrial generation of superoxide and hydrogen peroxide as the source of mitochondrial redox signaling. Free Radic. Biol. Med..

[40] Quinlan C.L., Orr A.L., Perevoshchikova I.V., Treberg J.R., Ackrell B.A., Brand M.D. (2012). Mitochondrial complex II can generate reactive oxygen species at high rates in both the forward and reverse reactions. J. Biol. Chem..

[41] Siebels I., Dröse S. (2013). Q-site inhibitor induced ROS production of mitochondrial complex II is attenuated by TCA cycle dicarboxylates. Biochim. Biophys. Acta-Bioenerg..

[42] Hinkle P.C., Butow R.A., Rackers E. (1967). Partial Resolution Phosphorylation. J. Biol. Chem..

[43] Chouchani E.T., Pell V.R., Gaude E., Aksentijević D., Sundier S.Y., Robb E.L., Logan A., Nadtochiy S.M., Ord E.N.J., Smith A.C. (2014). Ischaemic accumulation of succinate controls reperfusion injury through mitochondrial ROS. Nature.

[44] Ackrell B.A.C., Kearney E.B., Mayr M. (1974). Role of oxalacetate in the regulation of mammalian succinate dehydrogenase. J. Biol. Chem..

[45] Lambert A.J., Buckingham J.A., Boysen H.M., Brand M.D. (2008). Diphenyleneiodonium acutely inhibits reactive oxygen species production by mitochondrial complex I during reverse, but not forward electron transport. Biochim. Biophys. Acta-Bioenerg..

[46] Doughan A.K., Harrison D.G., Dikalov S.I. (2008). Molecular mechanisms of angiotensin II-mediated mitochondrial dysfunction: Linking mitochondrial oxidative damage and vascular endothelial dysfunction. Circ. Res..

[47] Andrukhiv A., Costa A.D., West I.C., Garlid K.D. (2006). Opening mitoKATP increases superoxide generation from complex I of the electron transport chain. Am. J. Physiol.-Hear. Circ. Physiol..

[48] Cobley J.N. (2020). Mechanisms of Mitochondrial ROS in Assisted Reproduction: The Known, the Unknown, and the Intriguing. Antioxidants.

[49] Nolfi-Donegan D., Braganza A., Shiva S. (2020). Mitochondrial electron transport chain: Oxidative phosphorylation, oxidant production, and methods of measurement. Redox Biol..

[50] Zorov D.B., Juhaszova M., Sollott S.J. (2014). Mitochondrial Reactive Oxygen Species (ROS) and ROS-Induced ROS Release. Physiol. Rev..

[51] Rivera J., Sobey C.G., Walduck A.K., Drummond G.R. (2010). Nox isoforms in vascular pathophysiology: Insights from transgenic and knockout mouse models. Redox Rep..

[52] Nickel A., Kohlhaas M., Maack C. (2014). Mitochondrial reactive oxygen species production and elimination. J. Mol. Cell. Cardiol..

[53] Dikalov S. (2011). Cross talk between mitochondria and NADPH oxidases. Free Radic. Biol. Med..

[54] Dikalov S.I., Dikalova A.E., Bikineyeva A.T., Schmidt H.H.H.W., Harrison D.G., Griendling K.K. (2008). Distinct roles of Nox1 and Nox4 in basal and angiotensin II-stimulated superoxide and hydrogen peroxide production. Free Radic. Biol. Med..

[55] Takac I., Schröder K., Zhang L., Lardy B., Anilkumar N., Lambeth J.D., Shah A.M., Morel F., Brandes R.P. (2011). The E-loop is involved in hydrogen peroxide formation by the NADPH oxidase Nox4. J. Biol. Chem..

[56] Block K., Gorin Y., Abboud H.E. (2009). Subcellular localization of Nox4 and regulation in diabetes. Proc. Natl. Acad. Sci. USA.

[57] Kuroda J., Ago T., Matsushima S., Zhai P., Schneider M.D., Sadoshima J. (2010). NADPH oxidase 4 (Nox4) is a major source of oxidative stress in the failing heart. Proc. Natl. Acad. Sci. USA.

[58] Canugovi C., Stevenson M.D., Vendrov A.E., Hayami T., Robidoux J., Xiao H., Zhang Y.Y., Eitzman D.T., Runge M.S., Madamanchi N.R. (2019). Increased mitochondrial NADPH oxidase 4 (NOX4) expression in aging is a causative factor in aortic stiffening. Redox Biol..

[59] Ago T., Kuroda J., Pain J., Fu C., Li H., Sadoshima J. (2010). Upregulation of Nox4 by hypertrophic stimuli promotes apoptosis and mitochondrial dysfunction in cardiac myocytes. Circ. Res..

[60] Lavoie J.L., Sigmund C.D. (2003). Minireview: Overview of the renin-angiotensin system-An endocrine and paracrine system. Endocrinology.

[61] Seshiah P.N., Weber D.S., Rocic P., Valppu L., Taniyama Y., Griendling K.K. (2002). Angiotensin II stimulation of NAD(P)H oxidase activity: Upstream mediators. Circ. Res..

[62] Youdim M.B.H., Edmondson D., Tipton K.F. (2006). The therapeutic potential of monoamine oxidase inhibitors. Nat. Rev. Neurosci..

[63] Menazza S., Blaauw B., Tiepolo T., Toniolo L., Braghetta P., Spolaore B., Reggiani C., di Lisa F., Bonaldo P., Canton M. (2010). Oxidative stress by monoamine oxidases is causally involved in myofiber damage in muscular dystrophy. Hum. Mol. Genet..

[64] Patole M.S., Swaroop A., Ramasarma T. (1986). Generation of H2O2 in Brain Mitochondria. J. Neurochem..

[65] Hauptmann N., Grimsby J., Shih J.C., Cadenas E. (1996). The metabolism of tyramine by monoamine oxidase A/B causes oxidative damage to mitochondrial DNA. Arch. Biochem. Biophys..

[66] Starkov A.A., Fiskum G., Chinopoulos C., Lorenzo B.J., Browne S.E., Patel M.S., Beal M.F. (2004). Mitochondrial α-ketoglutarate dehydrogenase complex generates reactive oxygen species. J. Neurosci..

[67] Fang H., Chen M., Ding Y., Shang W., Xu J., Zhang X., Zhang W., Li K., Xiao Y., Gao F. (2011). Imaging superoxide flash and metabolism-coupled mitochondrial permeability transition in living animals. Cell Res..

[68] Wang W., Fang H., Groom L., Cheng A., Zhang W., Liu J., Wang X., Li K., Han P., Zheng M. (2008). Superoxide Flashes in Single Mitochondria. Cell.

[69] Goncalves R.L.S., Watson M.A., Wong H.S., Orr A.L., Brand M.D. (2020). The use of site-specific suppressors to measure the relative contributions of different mitochondrial sites to skeletal muscle superoxide and hydrogen peroxide production. Redox Biol..

[70] Brand M.D., Goncalves R.L.S., Orr A.L., Vargas L., Gerencser A.A., Borch Jensen M., Wang Y.T., Melov S., Turk C.N., Matzen J.T. (2016). Suppressors of Superoxide-H2O2 Production at Site IQ of Mitochondrial Complex I Protect against Stem Cell Hyperplasia and Ischemia-Reperfusion Injury. Cell Metab..

[71] Orr A.L., Vargas L., Turk C.N., Baaten J.E., Matzen J.T., Dardov V.J., Attle S.J., Li J., Quackenbush D.C., Goncalves R.L.S. (2015). Suppressors of superoxide production from mitochondrial complex III. Nat. Chem. Biol..

[72] Goncalves R.L.S., Quinlan C.L., Perevoshchikova I.V., Hey-Mogensen M., Brand M.D. (2015). Sites of superoxide and hydrogen peroxide production by muscle mitochondria assessed ex vivo under conditions mimicking rest and exercise. J. Biol. Chem..

[73] Brand M.D. (2010). The sites and topology of mitochondrial superoxide production. Exp. Gerontol..

[74] Murphy M.P. (2009). How Mitochondria Produce Reactive Oxygen Species. J. Biochem..

[75] Mailloux R.J., Treberg J.R. (2016). Protein S-glutathionlyation links energy metabolism to redox signaling in mitochondria. Redox Biol..

[76] Edeas M., Weissig V. (2013). Targeting mitochondria: Strategies, innovations and challenges. The future of medicine will come through mitochondria. Mitochondrion.

[77] Murphy M.P., Hartley R.C. (2018). Mitochondria as a therapeutic target for common pathologies. Nat. Rev. Drug Discov..

[78] Serviddio G., Bellanti F., Sastre J., Vendemiale G., Altomare E. (2010). Targeting Mitochondria: A New Promising Approach for the Treatment of Liver Diseases. Curr. Med. Chem..

[79] Ross M.F., Kelso G.F., Blaikie F.H., James A.M., Cocheme H.M., Filipovska A., Ros T.D., Hurd T.R., Smith R.A.J., Murphy M.P. (2005). Lipophilic triphenylphosphonium cations as tools in mitochondrial bioenergetics and free radical biology. Biokhimiya.

[80] Sheu S.S., Nauduri D., Anders M.W. (2006). Targeting antioxidants to mitochondria: A new therapeutic direction. Biochim. Biophys. Acta - Mol. Basis Dis..

[81] Cheng A.J., Bruton J.D., Lanner J.T., Westerblad H. (2015). Antioxidant treatments do not improve force recovery after fatiguing stimulation of mouse skeletal muscle fibres. J. Physiol..

[82] Asin-Cayuela J., Manas A.R.B., James A.M., Smith R.A.J., Murphy M.P. (2004). Fine-tuning the hydrophobicity of a mitochondria-targeted antioxidant. FEBS Lett..

[83] Skulachev V.P., Antonenko Y.N., Cherepanov D.A., Chernyak B.V., Izyumov D.S., Khailova L.S., Klishin S.S., Korshunova G.A., Lyamzaev K.G., Pletjushkina O.Y. (2010). Prevention of cardiolipin oxidation and fatty acid cycling as two antioxidant mechanisms of cationic derivatives of plastoquinone (SkQs). Biochim. Biophys. Acta-Bioenerg..

[84] Murphy M.P. (2016). Understanding and preventing mitochondrial oxidative damage. Biochem. Soc. Trans..

[85] Plotnikov E.Y., Zorov D.B. (2019). Pros and cons of use of mitochondria-targeted antioxidants. Antioxidants.

[86] Zielonka J., Joseph J., Sikora A., Hardy M., Ouari O., Vasquez-Vivar J., Cheng G., Lopez M., Kalyanaraman B. (2017). Mitochondria-Targeted Triphenylphosphonium-Based Compounds: Syntheses, Mechanisms of Action, and Therapeutic and Diagnostic Applications. Chem. Rev..

[87] Bentinger M., Tekle M., Dallner G. (2010). Coenzyme Q - Biosynthesis and functions. Biochem. Biophys. Res. Commun..

[88] Sohal R.S., Kamzalov S., Sumien N., Ferguson M., Rebrin I., Heinrich K.R., Forster M.J. (2006). Effect of coenzyme Q10 intake on endogenous coenzyme Q content, mitochondrial electron transport chain, antioxidative defenses, and life span of mice. Free Radic Biol Med..

[89] Martelli A., Testai L., Colletti A., Cicero A.F.G. (2020). Coenzyme Q10: Clinical applications in cardiovascular diseases. Antioxidants.

[90] Smith R.A.J., Porteous C.M., Gane A.M., Murphy M.P. (2003). Delivery of bioactive molecules to mitochondria in vivo. Proc. Natl. Acad. Sci. USA.

[91] Murphy M.P., Smith R.A.J. (2007). Targeting antioxidants to mitochondria by conjugation to lipophilic cations. Annu. Rev. Pharmacol. Toxicol..

[92] James A.M., Cochemé H.M., Smith R.A.J., Murphy M.P. (2005). Interactions of mitochondria-targeted and untargeted ubiquinones with the mitochondrial respiratory chain and reactive oxygen species: Implications for the use of exogenous ubiquinones as therapies and experimental tools. J. Biol. Chem..

[93] Maroz A., Anderson R.F., Smith R.A.J., Murphy M.P. (2009). Reactivity of ubiquinone and ubiquinol with superoxide and the hydroperoxyl radical: Implications for in vivo antioxidant activity. Free Radic. Biol. Med..

[94] Espinosa-García J. (2004). Theoretical Study of the Trapping of the OOH Radical by Coenzyme Q. J. Am. Chem. Soc..

[95] James A.M., Smith R.A.J., Murphy M.P. (2004). Antioxidant and prooxidant properties of mitochondrial Coenzyme Q. Arch. Biochem. Biophys..

[96] Nohl H., Kozlov A.V., Staniek K., Gille L. (2001). The multiple functions of coenzyme q. Bioorg. Chem..

[97] Nohl H., Gille L., Kozlov A.V. (1998). Antioxidant-derived prooxidant formation from ubiquinol. Free Radic. Biol. Med..

[98] Skulachev V.P. (2011). SkQ1 treatment and food restriction - two ways to retard an aging program of organisms. Aging (Albany. NY)..

[99] Anisimov V.N., Egorov M.V., Krasilschchikova M.S., Lyamzaev K.G., Manskikh V.A., Moshkin M.P., Novikov E.A., Popovich I.G., Rogovin K.A., Shabalina I.G. (2011). Effects of the Mitochondria-Targeted Antioxidant SkQ1 on Lifespan of Rodents. Aging (Albany. NY)..

[100] Antonenko Y.N., Avetisyan A.V., Bakeeva L.E., Chernyak B.V., Chertkov V.A., Domnina L.V., Ivanova O.Y., Izyumov D.S., Khailova L.S., Klishin S.S. (2008). Mitochondria-targeted plastoquinone derivatives as tools to interrupt execution of the aging program. 1. Cationic plastoquinone derivatives: Synthesis and in vitro studies. Biochemistry.

[101] Feniouk B.A., Skulachev M. (2018). SkQ1: The Road from Laboratory Bench to the Market. Mitochondrial Biol. Exp. Ther..

[102] Skulachev V.P. (2007). A biochemical approach to the problem of aging: “megaproject” on membrane-penetrating ions. the first results and prospects. Biochemistry.

[103] Neroev V.V., Archipova M.M., Bakeeva L.E., Fursova A.Z., Grigorian E.N., Grishanova A.Y., Iomdina E.N., Ivashchenko Z.N., Katargina L.A., Khoroshilova-Maslova I.P. (2008). Mitochondria-targeted plastoquinone derivatives as tools to interrupt execution of the aging program. 4. Age-related eye disease. SkQ1 returns vision to blind animals. Biochemistry.

[104] Skulachev V.P., Anisimov V.N., Antonenko Y.N., Bakeeva L.E., Chernyak B.V., Erichev V.P., Filenko O.F., Kalinina N.I., Kapelko V.I., Kolosova N.G. (2009). An attempt to prevent senescence: A mitochondrial approach. Biochim. Biophys. Acta-Bioenerg..

[105] Zhao K., Zhao G.M., Wu D., Soong Y., Birk A.V., Schiller P.W., Szeto H.H. (2004). Cell-permeable peptide antioxidants targeted to inner mitochondrial membrane inhibit mitochondrial swelling, oxidative cell death, and reperfusion injury. J. Biol. Chem..

[106] Feniouk B.A., Skulachev V.P. (2017). Cellular and Molecular Mechanisms of Action of Mitochondria-Targeted Antioxidants. Curr. Aging Sci..

[107] Szeto H.H. (2014). First-in-class cardiolipin-protective compound as a therapeutic agent to restore mitochondrial bioenergetics. Br. J. Pharmacol..

[108] Szeto H.H., Schiller P.W. (2011). Novel therapies targeting inner mitochondrial membrane-from discovery to clinical development. Pharm. Res..

[109] Reddy T.P., Manczak M., Calkins M.J., Mao P., Reddy A.P., Shirendeb U., Park B., Hemachandra Reddy P. (2011). Toxicity of neurons treated with herbicides and neuroprotection by mitochondria-targeted antioxidant SS31. Int. J. Environ. Res. Public Health.

[110] Li J., Chen X., Xiao W., Ma W., Li T., Huang J., Liu X., Liang X., Tang S., Luo Y. (2011). Mitochondria-targeted antioxidant peptide SS31 attenuates high glucose-induced injury on human retinal endothelial cells. Biochem. Biophys. Res. Commun..

[111] Cho J., Won K., Wu D.L., Soong Y., Liu S., Szeto H.H., Hong M.K. (2007). Potent mitochondria-targeted peptides reduce myocardial infarction in rats. Coron. Artery Dis..

[112] Cho S., Szeto H.H., Kim E., Kim H., Tolhurst A.T., Pinto J.T. (2007). A novel cell-permeable antioxidant peptide, SS31, attenuates ischemic brain injury by down-regulating CD36. J. Biol. Chem..

[113] Birk A.V., Chao W.M., Bracken C., Warren J.D., Szeto H.H. (2014). Targeting mitochondrial cardiolipin and the cytochrome c/cardiolipin complex to promote electron transport and optimize mitochondrial ATP synthesis. Br. J. Pharmacol..

[114] Cerrato C., Langel U. (2018). Cell Penetrating Peptides Targeting Mitochondria. Mitochondrial Biol. Exp. Ther..

[115] Kelso G.F., Maroz A., Cochemé H.M., Logan A., Prime T.A., Peskin A.V., Winterbourn C.C., James A.M., Ross M.F., Brooker S. (2012). A mitochondria-targeted macrocyclic Mn(II) superoxide dismutase mimetic. Chem. Biol..

[116] Stoyanovsky D.A., Jiang J., Murphy M.P., Epperly M., Zhang X., Li S., Greenberger J., Kagan V., Bayr H. (2014). Design and synthesis of a mitochondria-targeted mimic of glutathione peroxidase, mitoebselen-2, as a radiation mitigator. ACS Med. Chem. Lett..

[117] Simioni C., Zauli G., Martelli A.M., Vitale M., Gonelli A., Neri L.M. (2018). Oxidative stress: role of physical exercise and antioxidant nutraceuticals in adulthood and aging. Oncotarget.

[118] Fogarty M.C., Devito G., Hughes C.M., Burke G., Brown J.C., McEneny J., Brown D., McClean C., Davison G.W. (2013). Effects of α-lipoic acid on mtDNA damage after isolated muscle contractions. Med. Sci. Sports Exerc..

[119] Williamson J., Hughes C.M., Cobley J.N., Davison G.W. (2020). The mitochondria-targeted antioxidant MitoQ, attenuates exercise-induced mitochondrial DNA damage. Redox Biol..

[120] Cobley J.N., Margaritelis N.V., Morton J.P., Close G.L., Nikolaidis M.G., Malone J.K. (2015). The basic chemistry of exercise-induced DNA oxidation: Oxidative damage, redox signaling, and their interplay. Front. Physiol..

[121] Chatgilialoglu C., D’Angelantonio M., Kciuk G., Bobrowski K. (2011). New insights into the reaction paths of hydroxyl radicals with 2′-deoxyguanosine. Chem. Res. Toxicol..

[122] Dizdaroglu M., Jaruga P. (2012). Mechanisms of free radical-induced damage to DNA. Free Radic. Res..

[123] Illés E., Mizrahi A., Marks V., Meyerstein D. (2019). Carbonate-radical-anions, and not hydroxyl radicals, are the products of the Fenton reaction in neutral solutions containing bicarbonate. Free Radic. Biol. Med..

[124] Fleming A.M., Burrows C.J. (2020). On the irrelevancy of hydroxyl radical to DNA damage from oxidative stress and implications for epigenetics. Chem. Soc. Rev..

[125] Sakellariou G.K., Jackson M.J., Vasilaki A. (2014). Redefining the major contributors to superoxide production in contracting skeletal muscle. The role of NAD(P)H oxidases. Free Radic. Res..

[126] Pearson T., Kabayo T., Ng R., Chamberlain J., McArdle A., Jackson M.J. (2014). Skeletal muscle contractions induce acute changes in cytosolic superoxide, but slower responses in mitochondrial superoxide and cellular hydrogen peroxide. PLoS ONE.

[127] Michaelson L.P., Shi G., Ward C.W., Rodney G.G. (2010). Mitochondrial redox potential during contraction in single intact muscle fibers. Muscle and Nerve.

[128] Wang P., Li C.G., Qi Z., Cui D., Ding S. (2015). Acute exercise induced mitochondrial H2O2 production in mouse skeletal muscle: Association with p66Shc and FOXO3a signaling and antioxidant enzymes. Oxid. Med. Cell. Longev..

[129] Giorgio M., Migliaccio E., Orsini F., Paolucci D., Moroni M., Contursi C., Pelliccia G., Luzi L., Minucci S., Marcaccio M. (2005). Electron transfer between cytochrome c and p66Shc generates reactive oxygen species that trigger mitochondrial apoptosis. Cell.

[130] Dan Dunn J., Alvarez L.A.J., Zhang X., Soldati T. (2015). Reactive oxygen species and mitochondria: A nexus of cellular homeostasis. Redox Biol..

[131] Go Y.M., Chandler J.D., Jones D.P. (2015). The cysteine proteome. Free Radic. Biol. Med..

[132] Sies H. (2017). Hydrogen peroxide as a central redox signaling molecule in physiological oxidative stress: Oxidative eustress. Redox Biol..

[133] Starkov A.A., Andreyev A.Y., Zhang S.F., Starkova N.N., Korneeva M., Syromyatnikov M., Popov V.N. (2014). Scavenging of H2O2 by mouse brain mitochondria. J. Bioenerg. Biomembr..

[134] Munro D., Treberg J.R. (2017). A radical shift in perspective: Mitochondria as regulators of reactive oxygen species. J. Exp. Biol..

[135] Mailloux R.J. (2018). Mitochondrial antioxidants and the maintenance of cellular hydrogen peroxide levels. Oxid. Med. Cell. Longev..

[136] Murphy M.P. (2012). Mitochondrial thiols in antioxidant protection and redox signaling: Distinct roles for glutathionylation and other thiol modifications. Antioxidants Redox Signal..

[137] Finkel T. (2012). Signal transduction by mitochondrial oxidants. J. Biol. Chem..

[138] Powers S.K., Jackson M.J. (2008). Exercise-induced oxidative stress: Cellular mechanisms and impact on muscle force production. Physiol. Rev..

[139] Fogarty M.C., Hughes C.M., Burke G., Brown J.C., Trinick T.R., Duly E., Bailey D.M., Davison G.W. (2011). Exercise-induced lipid peroxidation: Implications for deoxyribonucleic acid damage and systemic free radical generation. Environ. Mol. Mutagen..

[140] Gomez-Cabrera M.C., Domenech E., Ji L.L., Viña J. (2006). Exercise as an antioxidant: It up-regulates important enzymes for cell adaptations to exercise. Sci. Sport..

[141] Slade L., Chalker J., Kuksal N., Young A., Gardiner D., Mailloux R.J. (2017). Examination of the superoxide/hydrogen peroxide forming and quenching potential of mouse liver mitochondria. Biochim. Biophys. Acta-Gen. Subj..

[142] Powers S.K., Ji L.L., Kavazis A.N., Jackson M.J. (2014). Reactive Oxygen Species: Impact on Skeletal Muscle. Crit. Rev. Biomed. Eng..

[143] Powers S.K., Radak Z., Ji L.L. (2016). Exercise-induced oxidative stress: Past, present and future. J. Physiol..

[144] García-Lepe U.O., Bermúdez-Cruz R.M. (2019). Mitochondrial Genome Maintenance: Damage and Repair Pathways. DNA Repair-An Updat..

[145] Pamplona R. (2011). Mitochondrial DNA damage and animal longevity: Insights from comparative studies. J. Aging Res..

[146] Yuan Y., Ju Y.S., Kim Y., Li J., Wang Y., Yoon C.J., Yang Y., Martincorena I., Creighton C.J., Weinstein J.N. (2020). Comprehensive molecular characterization of mitochondrial genomes in human cancers. Nat. Genet..

[147] Yao Y., Nishimura M., Murayama K., Kuranobu N., Tojo S., Beppu M., Ishige T., Itoga S., Tsuchida S., Mori M. (2019). A simple method for sequencing the whole human mitochondrial genome directly from samples and its application to genetic testing. Sci. Rep..

[148] Gonzalez-Hunt C.P., Wadhwa M., Sanders L.H. (2018). DNA damage by oxidative stress: Measurement strategies for two genomes. Curr. Opin. Toxicol..

[149] Cline S.D. (2012). Mitochondrial DNA damage and its consequences for mitochondrial gene expression. Biochim. Biophys. Acta - Gene Regul. Mech..

[150] Richter C., Park J.W., Ames B.N. (1988). Normal oxidative damage to mitochondrial and nuclear DNA is extensive. Proc. Natl. Acad. Sci. USA.

[151] Yakes F.M., Van Houten B. (1997). Mitochondrial DNA damage is more extensive and persists longer than nuclear DNA damage in human cells following oxidative stress. Proc. Natl. Acad. Sci. USA.

[152] Mikhed Y., Daiber A., Steven S. (2015). Mitochondrial oxidative stress, mitochondrial DNA damage and their role in age-related vascular dysfunction. Int. J. Mol. Sci..

[153] Souza-Pinto N.C., Croteau D.L., Hudson E.K., Hansford R.G., Bohr V.A. (1999). Age-associated increase in 8-oxo-deoxyguanosine glycosylase/AP lyase activity in rat mitochondria. Nucleic Acids Res..

[154] Barja G., Herrero A. (2000). Oxidative damage to mitochondrial DNA is inversely related to maximum life span in the heart and brain of mammals. FASEB J..

[155] Shokolenko I., Venediktova N., Bochkareva A., Wilson G.I., Alexeyev M.F. (2009). Oxidative stress induces degradation of mitochondrial DNA. Nucleic Acids Res..

[156] Saki M., Prakash A. (2017). DNA damage related crosstalk between the nucleus and mitochondria. Free Radic. Biol. Med..

[157] Bohovych I., Khalimonchuk O. (2016). Sending out an SOS: Mitochondria as a signaling hub. Front. Cell Dev. Biol..

[158] Da Cunha F.M., Torelli N.Q., Kowaltowski A.J. (2015). Mitochondrial Retrograde Signaling: Triggers, Pathways, and Outcomes. Oxid. Med. Cell. Longev..

[159] Barbour J.A., Turner N. (2014). Mitochondrial stress signaling promotes cellular adaptations. Int. J. Cell Biol..

[160] Lee H.C., Wei Y.H. (2005). Mitochondrial biogenesis and mitochondrial DNA maintenance of mammalian cells under oxidative stress. Int. J. Biochem. Cell Biol..

[161] Marcu R., Wiczer B.M., Neeley C.K., Hawkins B.J. (2014). Mitochondrial Matrix Ca2+ Accumulation Regulates Cytosolic NAD+/NADH Metabolism, Protein Acetylation, and Sirtuin Expression. Mol. Cell. Biol..

[162] Valavanidis A., Vlachogianni T., Fiotakis C. (2009). 8-Hydroxy-2′ -deoxyguanosine (8-OHdG): A critical biomarker of oxidative stress and carcinogenesis. J. Environ. Sci. Heal.-Part C Environ. Carcinog. Ecotoxicol. Rev..

[163] Potenza L., Calcabrini C., De Bellis R., Guescini M., Mancini U., Cucchiarini L., Nappo G., Alloni R., Coppola R., Dugo L. (2011). Effects of reactive oxygen species on mitochondrial content and integrity of human anastomotic colorectal dehiscence: A preliminary DNA study. Can. J. Gastroenterol..

[164] Schlame M., Greenberg M.L. (2017). Biosynthesis, remodeling and turnover of mitochondrial cardiolipin. Biochim. Biophys. Acta-Mol. Cell Biol. Lipids.

[165] Yin F., Sancheti H., Liu Z., Cadenas E. (2016). Mitochondrial function in ageing: Coordination with signalling and transcriptional pathways. J. Physiol..

[166] Wan M., Hua X., Su J., Thiagarajan D., Frostegård A.G., Haeggström J.Z., Frostegård J. (2014). Oxidized but not native cardiolipin has pro-inflammatory effects, which are inhibited by Annexin A5. Atherosclerosis.

[167] Di Domenico F., Tramutola A., Butterfield D.A. (2017). Role of 4-hydroxy-2-nonenal (HNE) in the pathogenesis of alzheimer disease and other selected age-related neurodegenerative disorders. Free Radic. Biol. Med..

[168] Li T., Zhang Z., Kolwicz S.C., Abell L., Roe N.D., Kim M., Zhou B., Cao Y., Ritterhoff J., Gu H. (2017). Defective Branched-Chain Amino Acid Catabolism Disrupts Glucose Metabolism and Sensitizes the Heart to Ischemia-Reperfusion Injury. Cell Metab..

[169] Yao J., Diaz Brinton R. (2011). Targeting Mitochondrial Bioenergetics for Alzheimers Prevention and Treatment. Curr. Pharm. Des..

[170] Ademowo O.S., Dias H.K.I., Burton D.G.A., Griffiths H.R. (2017). Lipid (per) oxidation in mitochondria: An emerging target in the ageing process?. Biogerontology.

[171] Basova L.V., Kurnikov I.V., Wang L., Ritov V.B., Belikova N.A., Vlasova I.I., Pacheco A.A., Winnica D.E., Peterson J., Bayir H. (2007). Cardiolipin switch in mitochondria: Shutting off the reduction of cytochrome c and turning on the peroxidase activity. Biochemistry.

[172] Shen Z., Ye C., McCain K., Greenberg M.L. (2015). The Role of Cardiolipin in Cardiovascular Health. Biomed Res. Int..

[173] Nomura K., Imai H., Koumura T., Kobayashi T., Nakagawa Y. (2000). Mitochondrial phospholipid hydroperoxide glutathione peroxidase inhibits the release of cytochrome c from mitochondria by suppressing the peroxidation of cardiolipin in hypoglycaemia-induced apoptosis. Biochem. J..

[174] Choi S.Y., Gonzalvez F., Jenkins G.M., Slomianny C., Chretien D., Arnoult D., Petit P.X., Frohman M.A. (2007). Cardiolipin deficiency releases cytochrome c from the inner mitochondrial membrane and accelerates stimuli-elicited apoptosis. Cell Death Differ..

[175] Gentile F., Arcaro A., Pizzimenti S., Daga M., Paolo Cetrangolo G., Dianzani C., Lepore A., Graf M., Ames R.J.P., Barrera G. (2017). DNA damage by lipid peroxidation products: implications in cancer, inflammation and autoimmunity. AIMS Genet..

[176] Winczura A., Zdzalik D., Tudek B. (2012). Damage of DNA and proteins by major lipid peroxidation products in genome stability. Free Radic. Res..

[177] Ayala A., Munoz M.F., Arguelles S. (2014). Lipid Peroxidation: Production, Metabolism, and Signaling Mechanisms of Malondialdehyde and 4-Hydroxyl-2-Nonenal. Oxid. Med. Cell. Longev..

[178] Fisher-Wellman K., Bloomer R.J., Rossman M.J., Santos-Parker J.R., Steward C.A.C., Bispham N.Z., Cuevas L.M., Rosenberg H.L., Woodward K.A., Chonchol M. (2010). Mitochondrial biology and experimental therapeutics. Free Radic. Biol. Med..

[179] Kelso G.F., Porteous C.M., Coulter C.V., Hughes G., Porteous W.K., Ledgerwood E.C., Smith R.A.J., Murphy M.P. (2001). Selective targeting of a redox-active ubiquinone to mitochondria within cells: Antioxidant and antiapoptotic properties. J. Biol. Chem..

[180] Petróczi A., Naughton D.P., Pearce G., Bailey R., Bloodworth A., McNamee M. (2008). Nutritional supplement use by elite young UK athletes: Fallacies of advice regarding efficacy. J. Int. Soc. Sports Nutr..

[181] Jäger R., Purpura M., Kerksick C.M. (2019). Eight weeks of a high dose of curcumin supplementation may attenuate performance decrements following muscle-damaging exercise. Nutrients.

[182] Morrison D., Hughes J., Della Gatta P.A., Mason S., Lamon S., Russell A.P., Wadley G.D. (2015). Vitamin C and e supplementation prevents some of the cellular adaptations to endurance-training in humans. Free Radic. Biol. Med..

[183] Reid M.B., Khawli F.A., Moody M.R. (1993). Reactive oxygen in skeletal muscle. III. Contractility of unfatigued muscle. J. Appl. Physiol..

[184] Reid M.B., Stokić D.S., Koch S.M., Khawli F.A., Leis A.A. (1994). N-acetylcysteine inhibits muscle fatigue in humans. J. Clin. Invest..

[185] Lamb G.D., Westerblad H. (2011). Acute effects of reactive oxygen and nitrogen species on the contractile function of skeletal muscle. J. Physiol..

[186] Mason S.A., Trewin A.J., Parker L., Wadley G.D. (2020). Antioxidant supplements and endurance exercise: Current evidence and mechanistic insights. Redox Biol..

[187] Merry T.L., Ristow M. (2016). Do antioxidant supplements interfere with skeletal muscle adaptation to exercise training?. J. Physiol..

[188] Reid M.B. (2016). Redox interventions to increase exercise performance. J. Physiol..

[189] Matuszczak Y., Farid M., Jones J., Lansdowne S., Smith M.A., Taylor A.A., Reid M.B. (2005). Effects of N-acetylcysteine on glutathione oxidation and fatigue during handgrip exercise. Muscle Nerve.

[190] McKenna M.J., Medved I., Goodman C.A., Brown M.J., Bjorksten A.R., Murphy K.T., Petersen A.C., Sostaric S., Gong X. (2006). N-acetylcysteine attenuates the decline in muscle Na+, K+-pump activity and delays fatigue during prolonged exercise in humans. J. Physiol..

[191] Siegel M.P., Kruse S.E., Percival J.M., Goh J., White C.C., Hopkins H.C., Kavanagh T.J., Szeto H.H., Rabinovitch P.S., Marcinek D.J. (2013). Mitochondrial-targeted peptide rapidly improves mitochondrial energetics and skeletal muscle performance in aged mice. Aging Cell.

[192] Williamson J., Hughes C.M., Davison G.W. (2018). Exogenous plant-based nutraceutical supplementation and peripheral cell mononuclear DNA damage following high intensity exercise. Antioxidants.

[193] de Moraes M.S., Guerreiro G., Sitta A., de Moura Coelho D., Manfredini V., Wajner M., Vargas C.R. (2020). Oxidative damage in mitochondrial fatty acids oxidation disorders patients and the in vitro effect of l-carnitine on DNA damage induced by the accumulated metabolites. Arch. Biochem. Biophys..

[194] Hayashi G., Cortopassi G. (2015). Oxidative Stress in Inherited Mitochondrial Diseases. Physiol. Behav..

[195] Liguori I., Russo G., Curcio F., Bulli G., Aran L., Della-Morte D., Gargiulo G., Testa G., Cacciatore F., Bonaduce D. (2018). Oxidative stress, aging, and diseases. Clin. Interv. Aging.

[196] Snow B.J., Rolfe F.L., Lockhart M.M., Frampton C.M., O’Sullivan J.D., Fung V., Smith R.A.J., Murphy M.P., Taylor K.M. (2010). A double-blind, placebo-controlled study to assess the mitochondria- targeted antioxidant MitoQ as a disease-modifying therapy in Parkinson’s disease. Mov. Disord..

[197] Gane E.J., Weilert F., Orr D.W., Keogh G.F., Gibson M., Lockhart M.M., Frampton C.M., Taylor K.M., Smith R.A.J., Murphy M.P. (2010). The mitochondria-targeted anti-oxidant mitoquinone decreases liver damage in a phase II study of hepatitis C patients. Liver Int..

[198] Karaa A., Haas R., Goldstein A., Vockley J., Douglas Weaver W., Cohen B.H. (2018). Randomized dose-escalation trial of elamipretide in adults with primary mitochondrial myopathy. Neurology.

[199] Shill D.D., Southern W.M., Willingham T.B., Lansford K.A., McCully K.K., Jenkins N.T. (2016). Mitochondria-Specific Antioxidant Supplementation does not Influence Endurance Exercise Training-Induced Adaptations in Circulating Angiogenic Cells, Skeletal Muscle Oxidative Capacity, or Maximal Oxygen Uptake. J. Physiol..

[200] Broome S.C., Woodhead J.S.T., Merry T.L. (2018). Mitochondria-targeted antioxidants and skeletal muscle function. Antioxidants.

[201] Min K., Smuder A.J., Kwon O.S., Kavazis A.N., Szeto H.H., Powers S.K. (2011). Mitochondrial-targeted antioxidants protect skeletal muscle against immobilization-induced muscle atrophy. J. Appl. Physiol..

[202] Ristow M., Zarse K. (2010). How increased oxidative stress promotes longevity and metabolic health: The concept of mitochondrial hormesis (mitohormesis). Exp. Gerontol..

[203] Handy D.E., Loscalzo J. (2012). Redox regulation of mitochondrial function. Antioxidants Redox Signal..

[204] Storozhevykh T.P., Senilova Y.E., Persiyantseva N.A., Pinelis V.G., Pomytkin I.A. (2007). Mitochondrial respiratory chain is involved in insulin-stimulated hydrogen peroxide production and plays an integral role in insulin receptor autophosphorylation in neurons. BMC Neurosci..

[205] Denu J.M., Tanner K.G. (1998). Specific and reversible inactivation of protein tyrosine phosphatases by hydrogen peroxide: Evidence for a sulfenic acid intermediate and implications for redox regulation. Biochemistry.

[206] Handy D.E., Lubos E., Yang Y., Galbraith J.D., Kelly N., Zhang Y.Y., Leopold J.A., Loscalzo J. (2009). Glutathione peroxidase-1 regulates mitochondrial function to modulate redox-dependent cellular responses. J. Biol. Chem..

[207] Maehama T., Dixon J.E. (1998). The Tumor Suppressor, PTEN /. J. Biol. Chem..

[208] Lee H.C., Yin P.H., Lu C.Y., Chi C.W., Wei Y.H. (2000). Increase of mitochondria and mitochondrial DNA in response to oxidative stress in human cells. Biochem. J..

[209] Lee H.C., Yin P.H., Chi C.W., Wei Y.H. (2002). Increase in mitochondrial mass in human fibroblasts under oxidative stress and during replicative cell senescence. J. Biomed. Sci..

[210] Piantadosi C.A., Suliman H.B. (2006). Mitochondrial transcription factor A induction by redox activation of nuclear respiratory factor 1. J. Biol. Chem..

[211] St-Pierre J., Drori S., Uldry M., Silvaggi J.M., Rhee J., Jäger S., Handschin C., Zheng K., Lin J., Yang W. (2006). Suppression of Reactive Oxygen Species and Neurodegeneration by the PGC-1 Transcriptional Coactivators. Cell.

[212] Valle I., Álvarez-Barrientos A., Arza E., Lamas S., Monsalve M. (2005). PGC-1α regulates the mitochondrial antioxidant defense system in vascular endothelial cells. Cardiovasc. Res..

[213] Alexeyev M., Shokolenko I., Wilson G., LeDoux S. (2013). The maintenance of mitochondrial DNA integrity - Critical analysis and update. Cold Spring Harb. Perspect. Biol..

[214] Zhao L. (2019). Mitochondrial DNA Degradation: A Quality Control Measure for Mitochondrial Genome Maintenance and Stress Response.

[215] Shokolenko I.N., Alexeyev M.F. (2015). Mitochondrial DNA: A disposable genome?. Biochim. Biophys. Acta - Mol. Basis Dis..

[216] Zhao L., Sumberaz P. (2020). Mitochondrial DNA Damage: Prevalence, Biological Consequence, and Emerging Pathways. Chem. Res. Toxicol..

[217] Schmitt M.W., Kennedy S.R., Salk J.J., Fox E.J., Hiatt J.B., Loeb L.A. (2012). Detection of ultra-rare mutations by next-generation sequencing. Proc. Natl. Acad. Sci. USA.

[218] Li R., Zhou P., Guo Y., Lee J.S., Zhou B. (2017). Tris (1, 3-dichloro-2-propyl) phosphate induces apoptosis and autophagy in SH-SY5Y cells: Involvement of ROS-mediated AMPK/mTOR/ULK1 pathways. Food Chem. Toxicol..

[219] Heid M.E., Keyel P.A., Kamga C., Shiva S., Watkins S.C., Salter R.D. (2013). Mitochondrial Reactive Oxygen Species Induces NLRP3-Dependent Lysosomal Damage and Inflammasome Activation. J. Immunol..

[220] Zhao R.Z., Jiang S., Zhang L., Yu Z. (2019). Bin Mitochondrial electron transport chain, ROS generation and uncoupling (Review). Int. J. Mol. Med..

[221] Burtscher J., Burtscher M., Millet G.P. (2020). Response to: The mitochondria-targeted antioxidant MitoQ attenuates exercise-induced mitochondrial DNA damage (Williamson et al., available online 6 August 2020, 101,673). Redox Biol..

